# *Tlr4* Deletion Modulates Cytokine and Extracellular Matrix Expression in Chronic Spinal Cord Injury, Leading to Improved Secondary Damage and Functional Recovery

**DOI:** 10.1523/JNEUROSCI.0778-23.2023

**Published:** 2024-02-07

**Authors:** Fari Ryan, Isaac Francos-Quijorna, Gerard Hernández-Mir, Catharine Aquino, Ralph Schlapbach, Elizabeth J. Bradbury, Samuel David

**Affiliations:** ^1^Centre for Research in Neuroscience and BRaIN Program, Research Institute of the McGill University Health Centre, Montreal, Quebec H3G 1A4, Canada; ^2^The Wolfson Centre for Age-Related Diseases, King’s College London, London SE1 1UL, United Kingdom; ^3^Centre for Immunobiology, Blizard Institute, Barts and The London School of Medicine, Queen Mary University of London, London E1 2AT, United Kingdom; ^4^Functional Genomics Center Zurich, ETH Zurich and University of Zurich, Zurich 8057, Switzerland

**Keywords:** cell death, chronic inflammation, cytokine, extracellular matrix molecules, locomotor recovery, serotonergic fiber sprouting, spinal cord injury, Toll-like receptor

## Abstract

Toll-like receptors (TLRs) play an important role in the innate immune response after CNS injury. Although TLR4 is one of the best characterized, its role in chronic stages after spinal cord injury (SCI) is not well understood. We examined the role of TLR4 signaling in injury-induced responses at 1 d, 7 d, and 8 weeks after spinal cord contusion injury in adult female TLR4 null and wild-type mice. Analyses include secondary damage, a range of transcriptome and protein analyses of inflammatory, cell death, and extracellular matrix (ECM) molecules, as well as immune cell infiltration and changes in axonal sprouting and locomotor recovery. Lack of TLR4 signaling results in reduced neuronal and myelin loss, reduced activation of NFκB, and decreased expression of inflammatory cytokines and necroptotic cell death pathway at a late time point (8 weeks) after injury. TLR4 null mice also showed reduction of scar-related ECM molecules at 8 weeks after SCI, accompanied by increase in ECM molecules associated with perineuronal nets, increased sprouting of serotonergic fibers, and improved locomotor recovery. These findings reveal novel effects of TLR4 signaling in chronic SCI. We show that TLR4 influences inflammation, cell death, and ECM deposition at late-stage post-injury when secondary injury processes are normally considered to be over. This highlights the potential for late-stage targeting of TLR4 as a potential therapy for chronic SCI.

## Significance Statement

Spinal cord injury (SCI) often results in life-long paralysis and sensory loss of the limbs. Much of the research on biological mechanisms in SCI is focused on the acute period after injury. However, most SCI patients have been living with their injuries for months or years. We now show a delayed effect of Toll-like receptor 4 (TLR4) mediated inflammation several months after injury that induces changes in cytokines, neuronal cell death and extracellular matrix deposition, and axonal sprouting that can influence functional recovery. This work addresses a question of much interest in the field of spinal cord injury and could also be of wider interest for recovery after brain trauma.

## Introduction

Spinal cord injury (SCI) leads to permanent loss of motor, sensory, and autonomic function below the level of lesion, often with chronic pain and other comorbidities (David and Lopez-Vales, 2021). Dysregulated inflammation, cell and tissue loss, tissue scarring and maladaptive changes in the extracellular matrix (ECM) contribute importantly to the failure of functional recovery following spinal cord trauma (David et al., 2012; Bradbury and Burnside, 2019; Tran et al., 2022). The initial injury to the spinal cord results in local damage to axons, neurons and glia, and tissue necrosis, which is rapidly followed by a period of aggressive inflammation associated with further cell death, tissue loss, tissue remodeling, and scarring, referred to as secondary damage (Silver and Miller, 2004; Bradbury and Burnside, 2019). Although inflammation after injury in many tissues is central to wound healing responses, which restores tissue homeostasis and promote repair, the neurotoxic inflammatory response after CNS injury fails to clear the necrotic tissue debris rapidly and does not initiate an effective tissue healing response (Schwab et al., 2014). This is highlighted for example in the slow rate of Wallerian degeneration in the CNS versus the peripheral nerves (Perrin et al., 2005; Vargas and Barres, 2007). This dysregulated inflammation causes long-term changes in the expression and deposition of ECM proteins, leading to fibrotic tissue remodeling, glial scarring, and the formation of cystic cavities (Tran et al., 2022). Although dysregulated inflammation and maladaptive synthesis of ECM molecules are thought to play central roles in SCI pathology, the underlying mechanisms that mediate acute and chronic events that lead to such changes are still not well understood.

Toll-like receptors (TLRs) are pattern recognition receptors that protect the host from pathogens. They are also involved in injury responses and contribute to sterile inflammation, tissue homeostasis, and wound repair (Ackermann et al., 1995; Owens, 2009; Heiman et al., 2014). They belong to a family of 12 or more TLRs in mice that are widely distributed in various tissues and cell types (Heiman et al., 2014). They have varied responses due to their capacity for multitasking (Kapurniotu et al., 2019), as well as depending on the subtype of TLRs, and the antigens (alarmins) they bind (Owens, 2009). In addition to immune cells at sites of CNS injury, TLRs are also expressed in microglia, astrocytes, oligodendrocytes, and neurons (Bsibsi et al., 2002; Bowman et al., 2003; Kigerl et al., 2007; Heiman et al., 2014). A wide range of “damage associated molecular patterns” (DAMPs) from endogenous tissue sources act as alarmins to regulate cytokine and glial responses to CNS injury (Owens, 2009; Yang et al., 2017). Our earlier proteomics analysis of SCI tissue showed that several ECM molecules including small proteoglycans found at the site of CNS injury act as alarmins to trigger inflammatory responses via TLR4 (Didangelos et al., 2016; Francos-Quijorna et al., 2022).

Although TLR4 is one of the best characterized TLRs, its role in SCI is complex and not fully understood (Kigerl et al., 2007; Church et al., 2016). The detrimental role of TLR4 signaling in inflammation in other contexts including neurodegeneration suggests the need for further studies (Owens, 2009; Hoogland et al., 2015; Huang et al., 2017). We have recently shown that TLR4 signaling in vitro is a key driver of chondroitin sulfate proteoglycan (CSPG)-mediated activation of a pro-inflammatory phenotype in bone marrow-derived macrophages and isolated microglia cultures, suggesting TLR4 might play a key role in preventing resolution of inflammation after SCI (Francos-Quijorna et al., 2022). In the current work, we used a mouse with a targeted deletion of the *TLR4* gene (Hoshino et al., 1999) to study the role of TLR4 signaling on molecular, cellular, and ECM changes in vivo, at acute (1 d), subacute (7 d), and chronic (8 weeks) time points after SCI. Seven days was chosen as an intermediate time point before the scar is formed and is the early period of resolution of inflammation (Francos-Quijorna et al., 2022), while 8 weeks is when the scar is well formed and much of the inflammation is resolved after SCI (Schwab and Bartholdi, 1996; Tran et al., 2018). Our study revealed unexpected late-stage effects of *TLR4* deletion on cell death pathways, chemokine/cytokine expression, NFκB signaling, ECM molecule expression, secondary damage, and functional recovery. This work highlights a novel role of TLR4 signaling in chronic SCI.

## Materials and Methods

### Mice

*TLR4^−/−^* (*Tlr4*^tm1Aki^) and *TLR4^+/+^* (wild-type littermates) on a C57BL/6 background were used for all experiments. *Tlr4*^tm1Aki^ mice were generated by Dr. Shizuo Akira (Osaka University; Hoshino et al., 1999) and obtained from Dr. Salman Qureshi (Research Institute of the McGill University Health Center) that were backcrossed onto a C57BL/6 background for over eight generations (Laplante et al., 2011) for work done in Montreal, Canada, or from the Francis Crick Institute (London, United Kingdom) for the flow cytometry work done in London, United Kingdom. Mice were on a standard mouse chow and water ad libitum and given environmental enrichment. They were housed in a specific pathogen-free facility in individually ventilated cages in a temperature- and humidity-controlled environment at 21.5°C on a 12 h light/dark cycle.

### Spinal cord surgery

Female mice (8–10 weeks of age) were deeply anesthetized with a mixture of ketamine/xylazine/acepromazine (50:5:1) and a single laminectomy made at thoracic vertebral 11 (T11), and then a moderate contusion injury of the spinal cord was made using the Infinite Horizon Impactor (Precision Scientific Instrumentation; force 50 kDyn and tissue displacement 400–600 µm) as described previously (Ghasemlou et al., 2005). After surgery, their bladders were manually emptied twice daily. All procedures including breeding, surgery, behavior testing and perfusions to collect tissue samples were approved by the McGill University Animal Care Committee and followed the guidelines of the Canadian Council on Animal Care. Animal procedures performed in London, United Kingdom, were done in accordance with the United Kingdom Animals (Surgical Procedures) Act 1986, approved by the Animal Welfare and Ethical Review Body (AWERB) of King's College London and conducted under Home Office Project License PEE6F3C82. *TLR4* knock-out and wild-type mice were used. We did not assess heterozygous mice as the experimental design already involved a large number of animals and groups involving severe in vivo SCI protocols. All animal work adhered to the ARRIVE guidelines (Percie du Sert et al., 2020).

### Locomotor analyses

Locomotor recovery was assessed using the 9-point Basso Mouse Scale (BMS; Basso et al., 2006). Two individuals trained in the Basso laboratory at Ohio State University scored the BMS locomotor behavior independently, and the consensus scores were recorded. These analyses were performed by observers blinded to experimental groups. In addition, locomotor recovery was also assessed at 8 weeks using the DigiGait system (Mouse Specifics) at a speed of 10 cm/s.

### Protein extraction and Western blotting

After intracardiac perfusion with PBS under deep anesthesia, ∼5 mm of the injured spinal cords centered on the lesion epicenter as we have done previously (Lopez-Vales et al., 2011; Kroner et al., 2014) were dissected out 7d and 8 weeks after SCI and equivalent regions for baseline evaluations in uninjured wild-type and *TLR4* null mice. Total protein was extracted with 1% Nonidet P-40 (Sigma-Aldrich), 1% sodium deoxycholate (Sigma-Aldrich), 2% SDS, 0.15 M sodium phosphate, pH 7.2, and 2 mM ethylenediaminetetraacetic acid (EDTA), containing a mixture of protease and phosphatase inhibitors (Roche Diagnostics) as described previously (Jeong and David, 2003). Nuclear proteins were extracted using a nuclear isolation kit (NT-032; Invent Biotechnologies) according to manufacturer's instruction. Extraction of extracellular proteins was performed in PBS-EDTA-perfused animals based on a method described by Didangelos et al. (2016). Briefly, spinal cord tissues were incubated with 0.08% SDS and 12.5 mM EDTA supplemented with proteinase inhibitor cocktail (P8340, Sigma-Aldrich) and mildly shaken for 4 h at room temperature (RT). After removing SDS solution, the samples were incubated in 4 M guanidine-HCl and 12.5 mM EDTA plus proteinase inhibitor cocktail and vortexed vigorously for 24 h at RT. Guanidine extract was mixed with 100% ethanol and incubated at −20°C for 16 h. Proteins were then precipitated with centrifugation (16,000 × *g* for 45 min), and the pellets were dried and redissolved in 0.2% SDS buffer. Protein concentrations were estimated using the Bio-Rad DC (catalog #500-0121). Twenty-five milligrams of proteins per sample were separated by SDS-polyacrylamide gel electrophoresis (SDS-PAGE) 4–12% Bis-Tris gels (for total and nuclear proteins) and 7% Tris-acetate gels (for ECM proteins; Invitrogen) transferred to polyvinylidene fluoride membrane (PVDF; Millipore). Membranes were blocked in 5% milk in 0.05% PBS–Tween 20 and incubated overnight at 4°C with primary antibodies against receptor-interacting protein kinase 3 (RIP3), mixed lineage kinase domain-like pseudokinase (MLKL), cleaved gasdermin D (GSDMD), myeloid differentiation primary response 88 (MyD88), matrix metalloproteinase-9 (MMP9), versican, lumican, phosphacan, decorin, collagen 1A1 (COL1A1), nuclear factor-kappa-B transcription complex (NFκB-P65), I kappa B alpha (IκBα), and phospho-IκBα. Details of suppliers, catalog numbers, and concentrations are provided in Table 1. Blots were washed and incubated with horseradish peroxidase-conjugated IgG (1:10,000–100,000; Jackson ImmunoResearch) and visualized with enhanced chemiluminescence (PerkinElmer). Equal loading of proteins was estimated by re-probing the membranes with the following antibodies: rabbit anti-Histone H3 (1:500; Abcam; ab1791) for nuclear protein, rabbit anti-β-actin (1:1,000; Sigma-Aldrich; A2066) for total proteins, and mouse anti-GAPDH (1:1,000; EMD Millipore; MAB374) for ECM proteins. The blots were quantified using ImageJ/Fiji software (version 2.3.0/1.53q, National Institutes of Health).

**Table 1.** Suppliers, catalog numbers, and concentrations of antibodies

Primary antibodies used for Western blotting Rabbit anti-phospho-RIP3 (1:500; Abcam; ab195117) Rabbit anti-phospho-MLKL (1:1,000; Abcam; ab196436) Rabbit anti-cleaved GSDMD (1:1,000; Abcam; ab255603) Goat anti-MyD88 (1:200; R&D Systems; AF3109) Rabbit anti-MMP9 (1:750; Abcam; ab38898) Mouse anti-versican (1:300; Invitrogen; MA5-27638) Goat anti-lumican (1:500; R&D Systems; AF2745) Rabbit anti-phosphacan (PTPRZ; 1:500; Invitrogen; PA5-101832) Rabbit anti-Decorin (1:500; Abcam; ab175404) Rabbit anti-COL1A1 (1:1,000; Cell Signaling Technology; 72026) Rabbit anti-NF-κB-P65 (1:1,000; Cell Signaling Technology; 8242) Rabbit anti-IκBα (1:1,000; Cell Signaling Technology; 4812) Mouse anti-phospho-IκBα (1:1,000; Cell Signaling Technology; 9246)
Primary antibodies used for immunofluorescence labeling to tissue sections Rabbit anti-phospho-MLKL (1:150; Abcam; ab196436) rabbit anti-TLR4 (1:100; Abclonal; A11226) Rabbit anti-MMP9 (1:150; EMD Millipore; AB19016) Rabbit anti-NFκB-P65 (1:400; Cell Signaling Technology; 8242) Biotin-conjugate WFA (1:500; Sigma-Aldrich; L1516) Mouse anti-CS-56 (1:200; Abcam; ab11570) Rabbit anti-aggrecan (1:200, EMD Millipore; AB1031) Mouse anti-versican (1:100; Invitrogen; MA5-27638) Goat anti-lumican (1:150; R&D Systems; AF2745) Rabbit anti-phosphocan (PTPRZ; 1:100; Invitrogen; PA5-101832) Rabbit anti-Decorin (1:300; Abcam; ab175404) Rat anti-serotonin (1:100; Abcam; ab6336)
Combined with the following cell type-specific antibodies
Rat anti-CD11b (for macrophages/microglia; 1:200; AbD Serotec; MCA711) Rat anti-glial fibrillary acidic protein (GFAP; for astrocytes; 1:500; Invitrogen; 13-0300) Rabbit anti-GFAP (for astrocytes; 1:1500; Agilent Dako; Z0334) Guinea pig anti-NeuN (for neurons; 1:400; Synaptic Systems; 266004) Mouse anti-NeuN (for neurons; 1:200; Invitrogen; MA5-33103) Mouse anti-CC1 (for oligodendrocytes; 1:200; EMD Millipore; OP80)

### Immunofluorescence and confocal microscopy

Under deep anesthesia, mice were perfused with phosphate buffer followed by 4% paraformaldehyde in 0.1 M phosphate buffer. After fixation in the same fixative for 24 h, the tissue was cryoprotected in 30% sucrose and 14-mm-thick cross sections of the spinal cord with a cryostat used for the immunofluorescence labeling. Tissue sections were incubated with 0.3% Triton X-100 (Sigma-Aldrich), 5% normal goat or donkey serum (Jackson ImmunoResearch), and 2% ovalbumin (Sigma-Aldrich) in PBS for 3 h at RT to block nonspecific binding of antibodies. Sections were then incubated overnight at 4°C with primary antibodies against TLR4, p-MLKL, NFκB, MMP9, *Wisteria floribunda* agglutinin (WFA), CS-56, aggrecan, versican, lumican, phosphocan, decorin, and serotonin (5-HT; see Table 1 for detailed information on antibodies). This was combined with cell type-specific antibodies also listed in Table 1. Sections were washed in 0.05% PBS–Tween 20 and incubated with ExtrAvidin-FITC (for WFA;1:500; Sigma-Aldrich; E2761) and appropriate fluorescence-conjugated secondary antibodies: donkey anti-rabbit Alexa Fluor 488, donkey anti-goat Alexa Fluor 568, donkey anti-guinea pig Alexa Fluor 647, donkey anti-rat Alexa Fluor 488, goat anti-mouse Alexa Fluor 488, goat anti-rabbit Alexa Fluor 488, goat anti-rat Alexa Fluor 568, goat anti-rabbit Alexa Fluor 647, and anti-guinea pig Alexa Fluor 568 (all 1:500, Invitrogen). Tissue sections were viewed with a confocal laser scanning microscope (FluoView FV1000, Olympus) and micrographs taken with the FV10-ASW 3.0 software (Olympus). Negative controls excluding primary antibodies were done and showed no staining. For comparing between groups, the same setting was applied in all images for each immunostaining. For quantification of immunofluorescence data, area of fluorescence and fluorescence intensity [as measured by Integrated Density (IntDen), which is the product of area and mean gray value] were quantified with ImageJ. For cell counts, confocal images of NeuN^+^ and CC1^+^ cells with DAPI-stained nuclei were obtained using a FluoView FV1000 microscope (Olympus). The number of NeuN^+^ cells in the entire cross section of the spinal cord was counted automatically using ImageJ, from one section per animal at each of the distances from the epicenter as indicated on the graph (*n* = 6 mice for NeuN and *n* = 5 mice for CC1). The number of CC1^+^ cells with DAPI-stained nuclei in the dorsal columns was counted manually at the epicenter and 500 µm on either side using ImageJ. The data were averaged and extrapolated to cells per square millimeter.

### Luxol fast blue staining and quantification

Luxol fast blue (LFB) staining was used to assess myelin loss in the whole cross section of the spinal cord. Sections were first dehydrated by immersing in graded ethanol solutions for 2 min each (50–95%) and then placed in a 0.1% LFB solution overnight at 37°C. The next day, after cooling the slides at 4°C for 1 h and then dipping in 95% ethanol, they were dipped in dH_2_O and incubated in 0.05% lithium carbonate solution for 5 min. After placing the slides in dH_2_O for 1 min, they were dehydrated in graded ethanol solutions (70–100%), placed in xylene three times for 5 min, mounted and coverslipped. Images of the whole cross sections were taken using Axioskop 2 Plus microscope (Carl Zeiss) using Bioquant image software (Bioquant Life Science). For quantification, the threshold feature of ImageJ was used to measure the area of spared myelin within an area of the whole cross section. The ratio of spared myelin to the whole cross-section area was measured at ∼200 μm intervals over a 2 mm length of the cord.

### Quantitative real-time polymerase chain reaction

Total RNA was extracted using RNeasy Mini Kit (Qiagen) following manufacturer's instructions. cDNA was reverse transcribed with the Qiagen QuantiNova Kit (catalog #205411) and amplified using an ABI StepOne cycler (Applied Biosystems) using specific primer pairs as indicated in Table 2 (all primers from iDT DNA) and Fast SYBR Green Master Mix (Applied Biosystems). Peptidylprolyl isomerase A (*ppia*) was used as an internal control gene. The results were quantified using the ΔΔCT method following standardization relative to *ppia* (Livak and Schmittgen, 2001).

**Table 2.** Primer sequences used for qRT-PCR

Gene	Forward sequence	Reverse sequence
*PPIA*	ATGTGCCAGGGTGGTGAC	GTTTGGTCCAGCATTTGCCC
*MLKL*	CCTGAAGGAGGCTAACCAGC	CTGGCTGGCTGACATCTGAA
*RIPK3*	TCAAGTTATGGCCTACTGGTGC	ATAGCCTTCACCTCCCAGGATA
*NLRP3*	TGCGTGTTCTCTGTATACCAC	ATCTTCAGCAGCAGCCCTTT
*MMP9*	CAAGCTGTGTAGGTAGCACATC	GTGGTATAGTGGGACACATAGTGG
*IL6*	GAGGATACCACTCCCAACAGACC	AAGTGCATCATCGTTGTTCATACA
*CCL3*	CCTCTGTCACCTGCTCAACA	GATGAATTGGCGTGGAATCT
*CXCL1*	GACCATGGCTGGGATTCACC	CCAAGGGAGCTTCAGGGTCA
*CXCL2*	CGCTGTCAATGCCTGAAGAC	ACACTCAAGCTCTGGATGTTCTTG
*TNFα*	TTCTCATTCCTGCTTGTGGC	ACAGGCTTGTCACTCGAATTTT
*MyD88*	GGACAAACGCCGGAACTTTT	ATTAGCTCGCTGGCAATGGA
*Il-1b*	ATGGGCAACCACTTACCTATTT	GTTCTAGAGAGTGCTGCCTAATG

### RNA sequencing library preparation

Quality of the isolated RNA was determined with a Fragment Analyzer (Agilent). Only samples with a 260 nm/280 nm ratio between 1.8 and 2.1 and a 28S/18S ratio within 1.5–2 were further processed. The TruSeq Stranded mRNA (Illumina) was used in the next steps. Briefly, total RNA samples (100–1000 ng) were poly-A-enriched and then reverse-transcribed into double-stranded cDNA. The cDNA samples were fragmented, end-repaired, and adenylated before ligation of TruSeq adapters containing unique dual indices (UDIs) for multiplexing. Fragments containing TruSeq adapters on both ends were selectively enriched by PCR. The quality and quantity of the enriched libraries were validated using a Fragment Analyzer (Agilent). The product was a smear with an average fragment size of ∼260 bp. The libraries were normalized to 10 nM in 10 mM Tris-Cl, pH 8.5, with 0.1% Tween 20.

### Cluster generation and sequencing

The NovaSeq 6000 (Illumina) was used for cluster generation and sequencing according to a standard protocol. Sequencing was paired end at 2× 150 bp or single end 100 bp.

### Count generation

Quality-based adapter trimming was executed with fastp with the options “–trim_front1 1 —trim_tail 1 –cut_tail 20 –trim_poly_x –poly_x_min_len 10 –length_required 18” (Chen et al., 2018). Filtered and trimmed sequences were aligned to the *Mus musculus* genome build from ENSEMBL (GRCm38.p5) using STAR (Dobin et al., 2013). Raw read counts were calculated with featureCounts of the Rsubread package (Liao et al., 2013).

After filtering for minimal expression, raw counts were voom transformed in order to fit to linear modeling, using a design matrix based on the six experimental groups [*TLR4* gene knock-out mice (*TLR4* KO) and wild-type littermate controls were used for each of the following three groups: naive (uninjured), 7 d, and 8 weeks after SCI; *n* = 8 female mice per group]. Differential expression analyses were conducted programmatically using R-4.1.1 on all sequences, using functions from the limma package (Ritchie et al., 2015). Statistical significance was calculated using a moderated *t* test with Benjamini–Hochberg correction for multiple comparisons (false discovery rate).

To minimize potential baseline differences between WT and *TLR4*^−/−^ mice, we normalized gene expression at 7 d and 8 weeks post-SCI by subtracting the average levels of each transcript in each group (WT or *TLR4*^−/−^), prior to analysis. Differentially expressed sequences were typically filtered by adjusted *p* value <0.05, mapped and later converted to Entrez ID and Symbol annotation. Pseudogenes were also removed prior to over-representation analyses (ORAs) and plotting Venn diagrams. Statistical enrichment was based on hypergeometric test and adjusted *p* values are displayed unless mentioned otherwise.

All plots were produced using different variations of the ggplot2 package (Wickham, 2016), and ORA pathway analyses were contrasted against KEGG (Kanehisa, 1997), Reactome (Gillespie et al., 2022), WikiPathways (Martens et al., 2021), and Gene Ontology (Mi et al., 2019) gene libraries, using DOSE (Yu et al., 2015) and clusterProfiler (Wu et al., 2021) libraries.

### Flow cytometry sample preparation

To study the dynamics of immune cells after SCI, we harvested spinal cords from female and male injured *TLR4* KO and wild-type control mice at days 1 and 7 after SCI from *TLR4* KO and WT mice (1 d, *n* = 3 males and 2 female *TLR4* KO; and *n* = 3 males and 2 females WT; 7 d, *n* = 1 male and 2 females *TLR4* KO; and *n* = 1 male and 3 females WT). Animals were deeply anaesthetized with sodium pentobarbital (Euthatal, 80 mg/kg, i.p.) and transcardially perfused with ice-cold 1× PBS + 2% EDTA. Immediately after perfusion, 8 mm of the injured spinal cord centered around the lesion epicenter was dissected and placed into ice-cold PBS. Tissue was mechanically dissociated and then passed through a 70 μm cell strainer (BD Falcon) and centrifuged at 300 × *g* at 4°C. The pellet was incubated with Myelin Removal Beads II (Miltenyi Biotec) and passed through LS Columns (Miltenyi Biotec) to elute cells.

### Fluorescence-activated cell sorting staining/gating and analysis

Cells isolated from the spinal cord 1 and 7 d after SCI were washed with cold PBS and then incubated with a live/dead stain (eBioscience). After cell counts, samples were incubated with anti-CD16 and CD32 (1:50, BD Biosciences) for 15 min on ice to block the Fc receptors and stained with specific extracellular antibodies for 30 min. For intracellular staining used in phenotype analysis experiments, cells were then washed, fixed using 2% PFA, and permeabilized with cell permeabilization buffer (Invitrogen) containing intracellular antibodies. Single-stained Compensation Beads (BD Biosciences) were used according to manufacturer's instructions to prepare compensation controls by incubating with fluorescently conjugated antibodies used in the experiments. Fluorescence minus one experiment and isotype-matched control samples were run prior to this study to establish the positiveness of the samples and to aid the optimization of the compensation matrix. Based on this, the compensation matrix was adjusted where necessary due to over- or under-compensation by the automated algorithm. Cells were acquired on LSRFortessa III flow cytometer (BD Biosciences), and the data were analyzed with FlowJo (V10, Treestar) software. Single live cells were gated on the basis of dead cell exclusion (L/D), side (SSC-A) and forward scatter (FSC-A) gating, and doublet exclusion using side scatter width (SSC-W) against SSC-A. To perform the analysis, cells were first gated for cluster of differentiation 45 (CD45) to ensure that only infiltrating leukocytes and resident microglia were selected. Then, a combination of markers was used to identify neutrophils, microglia, monocytes/macrophages, conventional dendritic cells 1(cDC1), cDC2, no-DC, CD4^+^, and CD8^+^ T-cells and β-cells (Table 3). The list of antibodies used for flow cytometry analysis is provided in Table 3. To study the phenotype of microglia and different monocyte/macrophage subsets, in addition to prior described antibodies, expression of CD206, MHC-II, iNOS, and Arg-I was evaluated. Data were analyzed with FlowJo (V10, Treestar) software, and population clustering was performed by t-distributed Stochastic Neighbor Embedding (t-SNE) FlowJo Plugin.

**Table 3.** Antibodies used for flow cytometry analysis

Combination of markers used to identify various cell population for flow cytometry Neutrophils: CD45^+^^high^, CD11b^+^^medium^, Ly6C^medium^, Ly6G^high^, CD3 and CD19^low^ and SSC^high^ Microglia: CD45^+^^low/medium^, CD11b^+^^medium/high^, Ly6C^low^, Ly6G^−^, CD11c^low^, CD3 and CD19^low^, and SSC^low/medium^ Monocytes/macrophages: CD45^+^^high^, CD11b^+^^medium/high^, Ly6C^variable^, Ly6G^−^, CD11c^low^, CD3 and CD19^low^, and SSC^low/medium^ cDC1: CD45+^high^, CD11b^+^^low/medium^, Ly6C^−^, Ly6G^−^, MHC-II^medium^, CD11c^high^, CD3 and CD19^low^, and SSC^low/medium^ cDC2: CD45^+^^high^, CD11b^+^^high^, Ly6C^−^, Ly6G^−^, MHC-II^high^, CD11c^high^, CD3 and CD19^low^, and SSC^low/medium^ mo-DC: CD45^+^^high^, CD11b^+^^high^, Ly6C^high^, Ly6G^−^, MHC-II^low/medium^, CD11c^high^, CD3 and CD19^low^, and SSC^low/medium^ CD4+ T-cells: CD45^+^^high^, CD11b^−^ or low, CD3^−^, CD4+, CD8^−^ CD8+ T-cells: CD45^+high^, CD11b^−^ or low, CD3^+^, CD4^−^, CD8^+^ β-cells: CD45^+^^high^, CD11b^−^ or low, CD11c^−^, CD3^−^, CD19^+^
Antibodies used in flow cytometry analysis CD45 (BUV395, 1:300, BD Biosciences) CD11b (BV421; 1:300; BD Biosciences) Ly6G (BUV737; 1:400; BD Biosciences) F4/80 (APC; 1:250; BD Biosciences) Ly6C (BV711; 1:300; BD Biosciences) CD11c (BV650; 1:250; BD Biosciences) MHC-II (FITC; 1:300; BD Biosciences) iNOS (PerCP; 1:250; BD Biosciences) ArgI (PE; 1:200; BD Biosciences) CD206 (PE-Cy7; 1:350; BD Biosciences) CD19 (BV605; 1:250; BD Biosciences) CD3 (AF700; 1:200; BD Biosciences) CD4 (APC-Cy7; 1:200; BD Biosciences) CD8 (PE-Dazzle594; 1:250; BD Biosciences) Live/dead UV (1:1,000; BioLegend) was used to discriminate dead cells

### t-SNE analysis

The complex maps of immune cells were plotted by t-SNE (Van der Maaten and Hinton, 2008), which reduced dimensionality of multicolor flow cytometry data into a two-dimensional data space (t-SNE-1 vs t-SNE-2). Concatenating graphs were generated from all samples in each group. Manually gated viable CD45^+^ leukocytes were overlaid into the t-SNE plots using FlowJo plugin (version v10-LLC) and clustered by relative marker expression into nodes using the following parameters: perplexity = 50, theta = 0.5, and 500 iterations.

### Blinding and randomization

Surgeries, experimental procedures, data collection, and all statistical analysis were completed with the investigator blind to the experimental coding.

### Statistical analyses

Data are shown as mean ± standard error of the mean (SEM). Statistical tests were performed using GraphPad Prism 8 and 9 software. Statistical analyses were performed by using Mann–Whitney *U* test, one-way ANOVA, or two-way repeated-measures ANOVA (RM-ANOVA) with *post hoc* Tukey’s or Bonferroni’s test for multiple comparisons. Differences were considered significant at *p* < 0.05.

## Results

### TLR4 null mice show improved neuronal survival, reduced myelin and oligodendrocyte loss, and changes in related cell death pathways in chronic SCI

**Figure 1.**
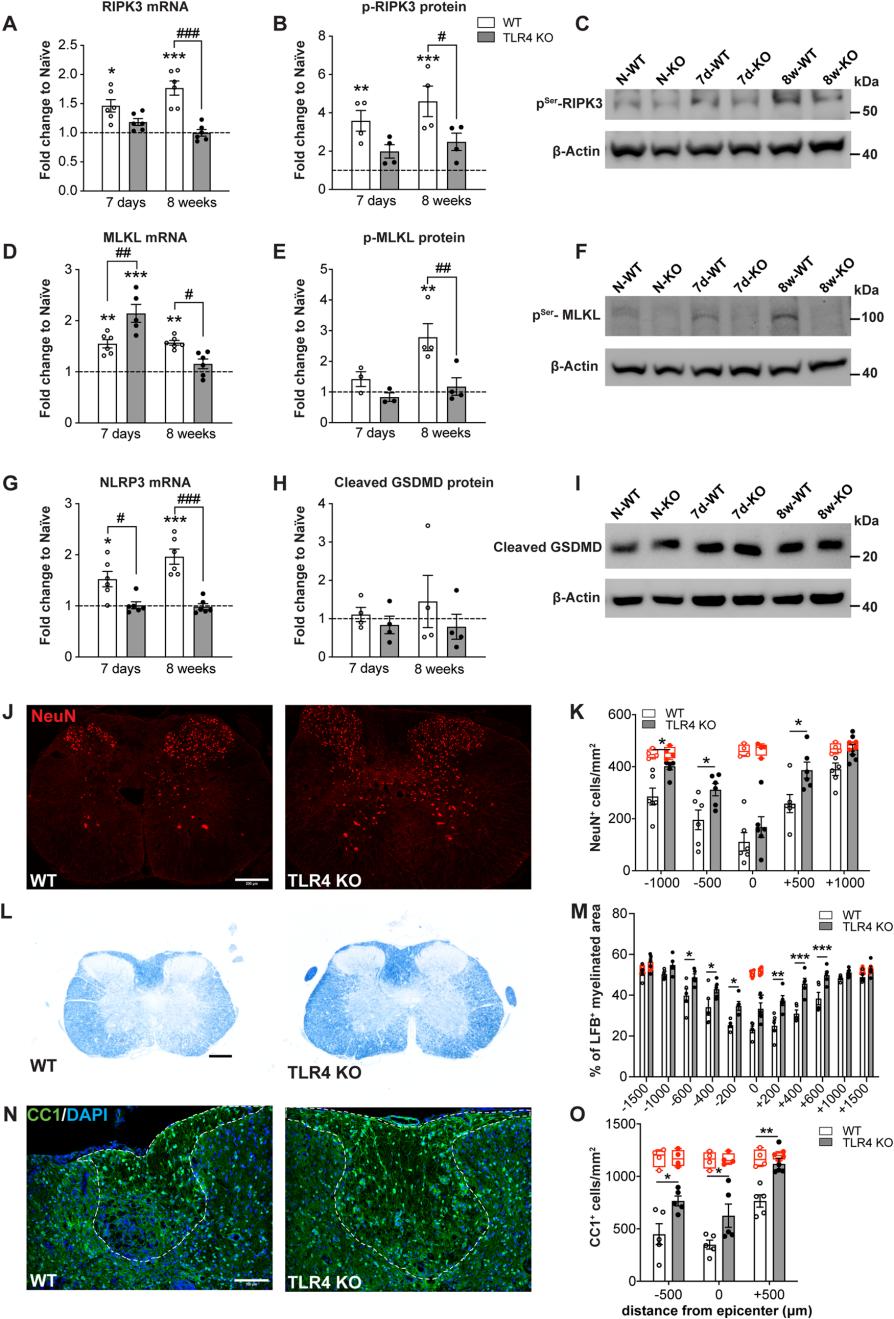
Improved neuronal survival, reduced myelin and oligodendrocyte loss, and changes in cell death pathways in chronic SCI in *TLR4* KO mice. ***A****–**C***, Changes in mRNA (***A***; *n* = 6 for all groups) and protein (***B***,***C***; *n* = 4 for all groups) in RIPK3 at 7 d and 8 weeks post-SCI in wild-type and *TLR4* null mice. Note the significantly lower mRNA and phospho-RIPK3 expression in *TLR4* null mice compared with wild-type mice at 8 weeks. Panel ***C*** shows Western blot. ***D–F***, Changes in mRNA (***D***; *n* = 5–6 for all groups) and protein (***E***,***F***; *n* = 3–4 for all groups expression of MLKL at 7 d and 8 weeks post-SCI in wild-type and *TLR4* null mice). Note again the significantly lower mRNA and phospho-MLKL protein expression in *TLR4* null mice compared with wild-type mice at 8 weeks. Each group normalized to its own controls. Panel ***F*** shows Western blot. ***G***, Changes in mRNA expression of NLRP3 at 7 d and 8 weeks post-SCI in wild-type and *TLR4* null mice. Note the significantly increased expression in wild-type mice at both time points, but no changes in *TLR4* null mice (*n* = 6 for all groups). ***H***, Quantification of Western blots shows no changes in expression of cleaved phospho-gasdermin D (GSDMD) at either time points in both genotypes (*n *= 3–4 for all groups). Panel ***I*** show Western blot. ***J***, Micrographs showing improved survival of NeuN^+^ neurons (500 µm rostral to lesion) in *TLR4* null mice compared with WT mice 8 weeks after SCI. ***K***, Quantification shows greater neuronal survival on either side of the lesion epicenter in TLR4 KO mice at 8 weeks post-SCI as detected by NeuN staining. The red boxplots show the values obtained from uninjured *TLR4* null and wild-type mice (*n* = 6 for all groups). ***L***, Micrographs of LFB staining shows reduced myelin loss (400 µm caudal to lesion) in *TLR4* null mice compared with WT mice 8 weeks after SCI. ***M***, Quantification of LFB staining shows greater myelin on either side of the lesion epicenter in *TLR4* KO mice. The red boxplots show the values obtained from uninjured *TLR4* null and wild-type mice (*n* = 5 for all groups). ***N***, CC1 staining of oligodendrocytes in the dorsal column of the spinal cord in WT and *TLR4* null mice. ***O***, Note increase in CC1^+^ oligodendrocytes in TLR4 null mice in the dorsal column white matter of the spinal cord compared with wild-type mice. The red boxplots show the values obtained from uninjured *TLR4* null and wild-type mice (*n* = 5 for all groups). One-way ANOVA with *post hoc* Tukey’s multiple-comparisons test (***A***,***B***,***D***,***E***,***G***,***H***); *p* < 0.0001 (***A***,***D***,***G***); *p* = 0.0001 (***B***); *p *= 0.001 (***E***); *p* = 0.807 (***H***). Two-way RM-ANOVA; genotype effect, with *post hoc* Bonferroni’s multiple-comparisons test (***K***,***M***,***O***); *p *= 0.008 (***K***); *p *= 0.0017 (***M***); *p *= 0.002 (***O***); *p ≤ 0.05; **p ≤ 0.01; ***p ≤ 0.001 compared with uninjured naive level. ^#^*p* ≤ 0.05; ^##^*p* ≤ 0.01; ^###^*p* ≤ 0.001 comparing the two injured genotypes. Scale bars: ***J***, ***L***, 200 µm; ***N***, 100 µm.

We first assessed if TLR4 signaling plays a role in inducing cell death through necroptosis via the adaptor protein TRIF engaging “receptor interacting protein kinase 3” (RIPK3) and “mixed lineage kinase domain-like protein” (MLKL; Humphries et al., 2015). Phosphorylation of MLKL and its translocation to the plasma membrane leads to loss of integrity of cell membranes and necroptosis-mediated cell death (Humphries et al., 2015). Necroptosis can lead to further release of DAMPs and continued cycle of TLR4 activation. We therefore assessed the level of RIPK3 (Fig. 1*A–C*) and MLKL (Fig. 1*D–F*) in the injured spinal cord at 7 d and 8 weeks after SCI by qRT-PCR and Western blotting. RIPK3 mRNA expression was significantly increased in injured wild-type spinal cord at 7 d and 8 weeks as compared with uninjured controls (Fig. 1*A*). The mRNA level in injured wild-type mice at 8 weeks was also significantly greater than that in *TLR4* KO mice (Fig. 1*A*). Importantly, RIPK3 mRNA levels were unchanged in injured *TLR4* null mice at both 7 d and 8 weeks (Fig. 1*A*; one-way ANOVA; *F*_(5,30)_ = 11.40; *p *< 0.0001; with *post hoc* Tukey’s multiple-comparisons test; naive vs 7d-WT, *p *= 0.018; naive vs 7d-KO, *p *= ns; naive vs 8w-WT, *p *< 0.0001; naive vs 8w-KO, *p *= ns; 7d-WT vs 7d-KO, *p *= ns; 8w-WT vs 8w-KO, *p *< 0.0001; *n *= 6 mice per group). Furthermore, Western blots show significant fourfold increase in phospho-RIPK3 in wild-type mice at 7 d and 8 weeks compared with uninjured controls (Fig. 1*B,C*). There was no statistically significant change in phopho-RIPK3 in the injured spinal cord of *TLR4* KO mice compared with uninjured controls (Fig. 1*B*). Notably, the level of phospho-RIPK3 was significantly greater in wild-type mice at 8 weeks compared with *TLR4* KO mice (Fig. 1*B*; one-way ANOVA; *F*_(5,18)_ = 9.71; *p *= 0.0001; with *post hoc* Tukey’s multiple-comparisons test; naive vs 7d-WT, *p *= 0.010; naive vs 7d-KO, *p *= ns; naive vs 8w-WT, *p *= 0.0004; naive vs 8w-KO, *p *= ns; 7d WT vs 7d-KO, *p *= ns; 8w-WT vs 8w-KO, *p *= 0.04; *n *= 4 mice per group). Phosphorylation of RIPK3 leads to recruitment of MLKL to the necrosome that triggers downstream necroptosis (Humphries et al., 2015). The mRNA levels of MLKL were increased at 7 d in both genotypes with the levels being significantly higher in *TRL4* null mice (Fig. 1*D*). At 8 weeks, however, MLKL mRNA was reduced to normal uninjured levels in *TLR4* null mice but remained significantly increased in wild-type mice compared with *TLR4* KO and uninjured controls (Fig. 1*D*; one-way ANOVA; *F*_(5,29)_ = 21.07; *p *< 0.0001; with *post hoc* Tukey’s multiple-comparisons test; naive vs 7d-WT, *p *= 0.002; naive vs 7d-KO, *p *< 0.0001; naive vs 8w-WT, *p *= 0.0016; naive vs 8w-KO, *p *= ns; 7d-WT vs 7d-KO, *p *= 0.002; 8w-WT vs 8w-KO, *p *= 0.03; *n *= 5–6 mice per group). Importantly, Western blots show that phospho-MLKL levels were unchanged in both genotypes at 7 d but significantly elevated (approximately threefold) at 8 weeks in wild-type but not in TLR4 KO mice (Fig. 1*E*,*F*; one-way ANOVA; *F*_(5,14)_ = 7.6; *p *= 0.001; with *post hoc* Tukey’s multiple-comparisons test; naive vs 8w-WT, *p *= 0.005; naive vs 8w-KO, *p *= ns; 7d-WT vs 7d-KO, *p *= ns; 8w-WT vs 8w-KO, *p *= 0.009; *n *= 3–4 mice per group). As phospho-MLKL levels are crucial to effect necroptosis, these results suggest a late effect in cell death that has not previously been recognized in SCI.

We also assessed markers of pyroptosis, a form of cell death associated with inflammation. Interestingly, the mRNA levels of NLRP3 are upregulated in wild-type mice at 7 d and 8 weeks after SCI but remains unchanged in *TLR4* null mice (Fig. 1*G*; one-way ANOVA; *F*_(5,30)_ = 13.60; *p *< 0.0001; with *post hoc* Tukey’s multiple-comparisons test; naive vs 7d-WT, *p *= 0.02; naive vs 7d-KO, *p *= ns; naive vs 8w-WT, *p *< 0.0001; naive vs 8w-KO, *p *= ns; 7d-WT vs 7d-KO, *p *= 0.02; 8w-WT vs 8w-KO, *p *< 0.0001; *n *= 6 mice per group). However, Western blots show that the pore-forming cleaved GSDMD which induces pyroptosis is not significantly different in *TLR4* null and wild-type mice at both time points after SCI as compared with uninjured controls (Fig. 1*H*,*I*; one-way ANOVA; *F*_(5,16)_ = 0.449; *p *= 0.807; with *post hoc* Tukey’s multiple-comparisons test; naive vs 7d-WT, *p *= ns; naive vs 7d-KO, *p *= ns; naive vs 8w-WT, *p *= ns; naive vs 8w-KO, *p *= ns; 7d-WT vs 7d-KO, *p *= ns; 8w-WT vs 8w-KO, *p *= ns; *n *= 3–4 mice per group). These results suggest that necroptosis but not pyroptosis is likely to contribute to cell death after SCI.

We next assessed whether *TLR4* deficiency leads to neuronal loss in the spinal cord 8 weeks after SCI. The number of NeuN^+^ neurons rostral and caudal to the lesion epicenter were significantly higher in *TLR4* null mice compared with injured wild-type mice (Fig. 1*J*,*K*), indicating reduced neuronal loss in *TLR4* null mice. This amounted to about a 35% increase in neuronal survival at 500 µm rostral and caudal to the lesion epicenter (two-way RM-ANOVA; genotype effect *F*_(1,10)_ = 10.56; *p *= 0.008; with *post hoc* Bonferroni’s multiple-comparisons test; *p*_(−1000)_ = 0.04; *p*_(−500)_ = 0.04; *p*_(+500)_ = 0.02; *n *= 6 mice per group), indicating reduced neuronal loss in *TLR4* null mice).

Myelin sparing was assessed in spinal cord cross sections stained with LFB. Myelin sparing was greater in the injured spinal cord of *TLR4* null mice as compared with wild-type mice 8 weeks after SCI (Fig. 1*L*,*M*). This difference was particularly notable at the lesion epicenter and regions rostral and caudal to the injury site (Fig. 1*M*; two-way RM-ANOVA; genotype effect *F*_(1,8)_ = 21.54; *p *= 0.0017; with *post hoc* Bonferroni’s multiple-comparisons test; *p*_(−600)_ = 0.04; *p*_(−400)_ = 0.04; *p*_(−200)_ = 0.02; *p*_(epicenter)_ = 0.012; *p*_(+200)_ = 0.0011; *p*_(+400)_ < 0.0001; *p*_(+600)_ = 0.003; *n *= 5 mice per group). Finally, quantification of CC1^+^ oligodendrocytes showed a significantly higher number in the dorsal column white matter of *TLR4* KO mice as compared with wild-type mice 8 weeks after SCI (Fig. 1*N*,*O*; two-way RM-ANOVA; genotype effect *F*_(1,8)_ = 19.85; *p *= 0.002; with *post hoc* Bonferroni’s multiple comparisons test; *p*_(−500)_ = 0.016; *p*_(epicenter)_ = 0.04; *p*_(+500)_ = 0.007; *n *= 5 mice per group).

### Cell type-specific localization of TLR4 and necroptosis marker after SCI

**Figure 2.**
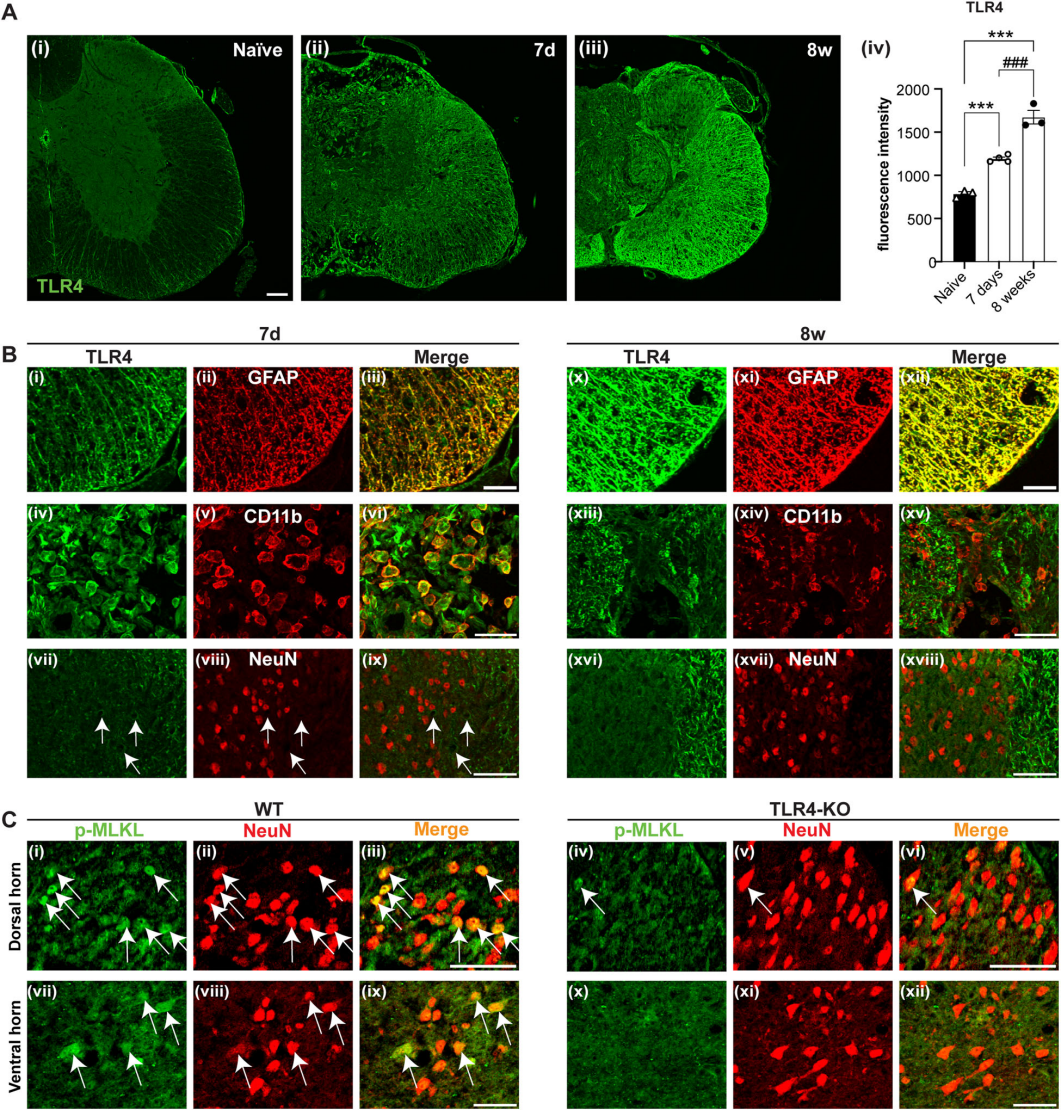
Immunostaining showing TLR4 expression at 7 d and 8 weeks after SCI and reduced expression of necroptosis marker (p-MLKL) at 8 weeks post-SCI. ***A***, TLR4 immunostaining of cross sections of the spinal cord of uninjured (naive; ***Ai***), 7 d (***Aii***), and 8 weeks (***Aiii***) after SCI. Note the increase in expression at 7 d which is further increased at 8 weeks. Note also in the 7 d panel (***Aii***) the elongated profiles of astrocyte-like cells in the lateral and ventrolateral white matter and rounded profile of macrophage-like cells in the dorsal region that sustains more damage. No staining was seen in negative controls (data not shown). The graph (***Aiv***) shows quantification of densitometric data of TLR4 staining (*n* = 3–4 for all groups); one-way ANOVA with *post hoc* Tukey’s multiple-comparisons test; *p* < 0.0001.) ***B***, Double immunofluorescence staining of spinal cord sections labeled with TLR4 (green) and either GFAP, CD11b, or NeuN (all red) for animals 7 d and 8 weeks after SCI. Note that the merged images show expression of TLR4 increases in astrocytes between 7 d (***Biii***) and 8 weeks (***Bxii***; ventrolateral white matter). TLR4 is expressed in CD11b^+^ macrophages at 7 d (***Bv***,***vi***) but not 8 weeks (***Bxiii***–***xv***). At the latter time point, note the TLR4 staining of CD11b-negative astrocyte-like profiles is seen (***Bxv***; dorsal region of the spinal cord). There is no expression of TLR4 in NeuN^+^ neurons (dorsal gray) at both time points (***Bvii***–***ix***,***xvi***–***xviii***). The rounded profiles outlined with weak TLR4 staining (arrows in ***Bvii***) are NeuN negative (***Bix***) indicating that these are likely to be profiles of blood vessels showing weak labeling of endothelial cells or astrocytes. Note, however, in the merged image (***Bxviii***) that TLR4 staining (green) is seen in the white matter along the right side. Sections for GFAP and CD11b taken close to the epicenter of the injury while for NeuN sections taken 500 µm caudal to the injury. ***C***, Double immunofluorescence staining of the dorsal horn region [***Ci***–***iii*** (WT) and ***Civ***–***vi*** (KO)] and ventral horn region [***Cvii***–***ix*** (WT) and ***Cx***–***xii*** (KO)] labeled with p-MLKL (green) and NeuN (red) and merge (yellow) at 8 weeks post-injury. Note that these merged images show increased expression of p-MLKL (yellow) in NeuN+ neurons in the dorsal horn (DH) and ventral horn (VH; arrows). This labeling is stronger in WT mice after SCI but shows sparse expression in NeuN^+^ neurons in *TLR4* null (KO) mice. Scale bars: ***A***, 100 µm; ***B***, ***C***, 50 µm.

To understand whether necroptosis-related changes in chronic SCI are mediated directly or indirectly via TLR4 signaling, we first assessed the expression of TLR4 in the uninjured spinal cord and in injured spinal cord at 7 d and 8 weeks after SCI. TLR4 expression detected by immunofluorescence is negligible in the uninjured spinal cord but increases 7 d after SCI and is increased even further at 8 weeks (Fig. 2*A*; one-way ANOVA; *F*_(2,7)_ = 91.8; *p *< 0.0001; with *post hoc* Tukey’s multiple-comparisons test; naive vs 7d-WT, *p *= 0.0008; naive vs 8w-WT, *p* < 0.0001; 7d-WT vs 8w-WT, *p *= 0.0003; *n *= 3–4 mice per group). Furthermore, double immunofluorescence labeling with cell type-specific markers showed that TLR4 is highly expressed in GFAP^+^ astrocytes at 7 d post-injury, with the expression increased even further at 8 weeks post-injury (Fig. 2*Biii*,*xii*). TLR4 expression in CD11b^+^ macrophages/microglia was observed only at 7 d post-injury but not at the chronic 8 week time point (Fig. 2*Bvi*–*xv*). We did not detect TLR4 staining in NeuN^+^ neurons at either time point (Fig. 2*Bvii*–*ix*,*xvi*–*xvii*). TLR4 staining appeared confined mainly to white matter regions (Fig. 2*Bvii*,*xvi*), while the small amount of weak TLR4 labeling in the gray matter was NeuN-negative and likely to be endothelial cells or astrocytes surrounding blood vessel profiles (Fig. 2*Bvii*,*ix*, arrows). We next assessed the spatial localization of the necroptosis marker (p-MLKL) in chronic SCI tissue from WT and *TLR4* null mutants. Double immunofluorescence staining revealed that at 8 weeks post-SCI, p-MLKL expression is increased in NeuN^+^ neurons in the dorsal and ventral gray matter (Fig. 2*C*). This staining is markedly reduced in *TLR4* KO mice (Fig. 2*C*). As TLR4 is not expressed in neurons, necroptosis in neurons is likely to be mediated indirectly via TLR4 signaling in non-neuronal cells.

### RNASeq analysis shows that TLR4 plays a remarkable role in ECM deposition and mitochondrial, synaptic, and neuronal functions at chronic stages after SCI

**Figure 3.**
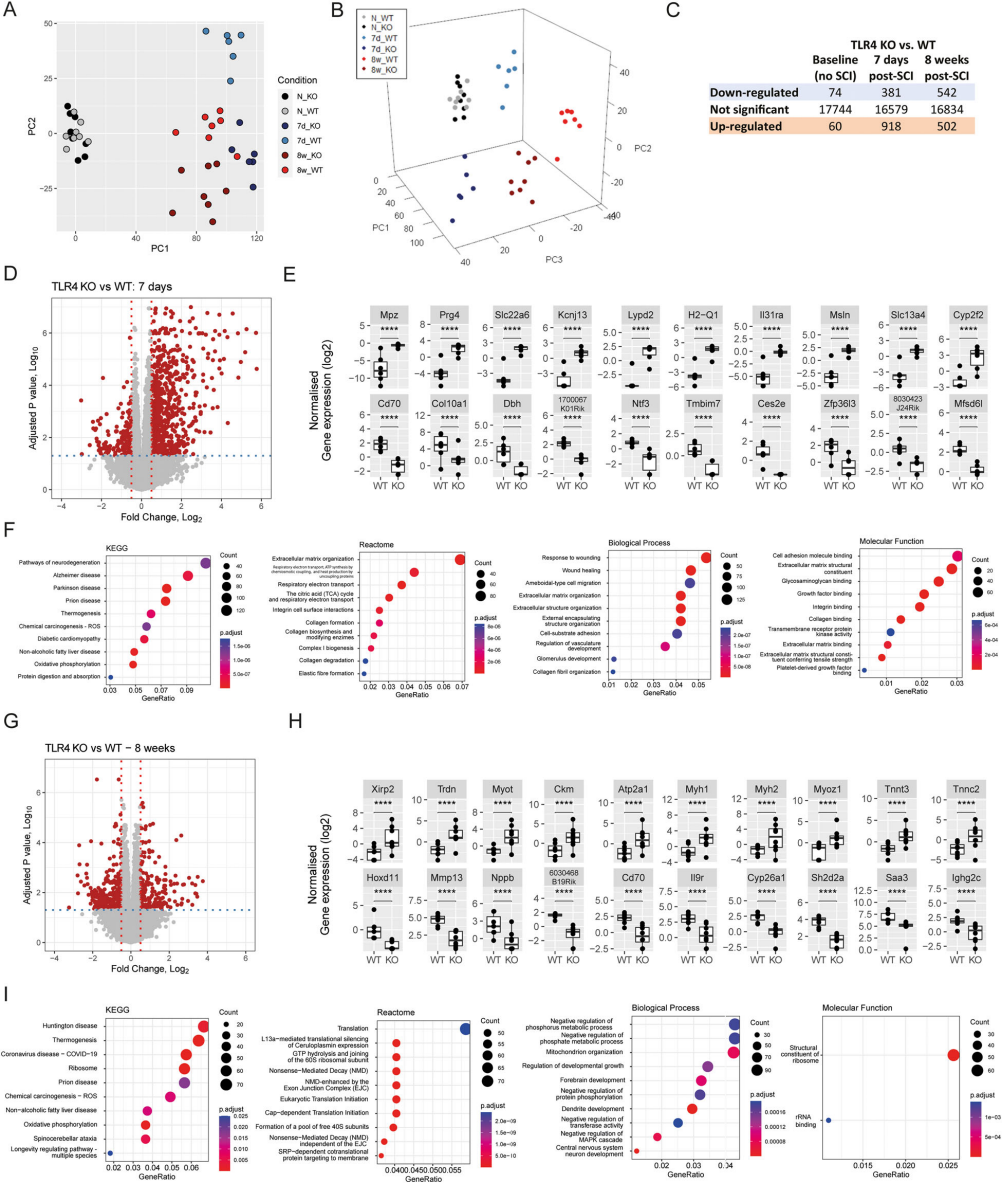
TLR4 null mice exhibit differential transcriptional responses at 7 d and 8 weeks post-SCI. 2D PCA (***A***) and 3D PCA (***B***) of bulk RNASeq filtered counts (17,878 sequences). PCA shows treatment separation along PC1, time point post intervention (PC3), and WT versus *TLR4* KO samples (PC2). ***C***, Summary of (sequence) pairwise differential expression analysis with six group design matrix between *TLR4^−/−^* and WT mice. Only differentially expressed sequences with adjusted *p* value (BH-correction) <0.05 were considered for downstream analyses. ***D***, Volcano plot showing statistical significance (adjusted *p* value) and log2 fold change (*TLR4* KO vs WT) at 7 d post-SCI. An adjusted *p* value threshold of 0.01 (dotted blue line) and an abs(log2FC) >0.25 (dotted red line) were used to highlight the most relevant differentially expressed sequences. From this pool, ***E*** shows the top 10 upregulated (sorted by log2FC; top) and the top 10 downregulated (sorted by log2FC; bottom) sequences between *TLR4* KO mice and WT at 7 d post-SCI. ***F***, ORA of 2,463 differentially expressed unique genes (*TLR4* KO vs WT) matching KEGG, Reactome, and Gene Ontology (Biological Process and Molecular Function) gene libraries. ***G***, Volcano plot and ***H*** top 10 up- and downregulated genes (sorted by log2FC) and (***I***) ORA pathway analysis in *TLR4* KO versus WT mice 8 weeks post-SCI. Significance scores are denoted as **p < 0.05, **p < 0.01, ***p < 0.001, and ****p < 0.0001.

To characterize the molecular effects of TLR4 deficiency in mice after SCI, we performed a bulk RNASeq from spinal cord samples taken at 7 d and 8 weeks after SCI. Pseudocounts were filtered for minimal expression in all samples and voom transformed to fit linear modeling. Data were validated to fit the expected mean–variance trend. We first determined whether there were basal differences between WT and *TLR4* KO naive (uninjured) mice and found minimal transcriptional differences (Fig. 3*A–C*). Importantly, these differences were marginal and over-representation pathway analysis revealed no pathways or functions enriched after multiple-comparisons correction (data not shown). However, to minimize any possible bias effects, and only evaluate the effect of SCI in each group, transcriptomic levels were normalized by subtracting the average transcript levels for uninjured WT and *TLR4*^−/−^ samples, respectively.

Principal component analysis (PCA) of all expressed sequences revealed that, transcriptionally, WT and *TLR4* KO mice are virtually identical prior to SCI (Fig. 3*A*,*B*). All other groups were clearly distinct in a three-dimensional PCA (Fig. 3*B*). As expected, the major contribution to explain the data variance was found in PC1 (60%) followed by PC2:PC3 (explaining up to 80% of all variances in the data; data not shown). Injured animals were markedly segregated from naive mice on principal component 1 (PC1), whereas PC3 separated samples at 7 d versus 8 weeks post-SCI and, more importantly, WT and *TLR4* KO samples clearly separate as independent groups on PC2, both at 7 d and 8 weeks post-SCI (Fig. 3*B*).

We further characterized the effects of TLR4 deficiency on differential gene expression at 7 d and 8 weeks post-SCI. At 7 d post-SCI, *TLR4* KO responses differed in ∼7% of the transcriptome, compared with WT responses (Fig. 3*C–F*). Most of the differentially expressed genes at 7 d were upregulated in the *TLR4* KO group (Fig. 3*C*,*D*). To visualize the most biologically relevant differences, we sorted the filtered differentially expressed genes (adjusted *p* value <0.01) by normalized gene expression (log2; Fig. 3*E*; TLR4^−/−^ vs WT, 7 d post-SCI). Analysis of the most differentially expressed genes (by FC) revealed differences in immune regulators (*Cd70*, *Il31ra*, *H2-Q1*, *Msln*), ECM components (*Prg4*), small molecule transporters (*Scl22a6*, *KCnj13*, *Scl13a4*, *Mfsd6l*), and cell death modulators (*Tmbim7*; Fig. 3*E*). Pathway analysis of the differences at 7 d post-SCI reveals enrichment in ECM deposition and hint at downstream or subsequent neurodegeneration (Fig. 3*F*). We next evaluated differential gene expression between *TLR4* KO and WT mice at 8 weeks post-SCI (Fig. 3*C*,*G–I*). At 8 weeks, *TLR4* KO responses differed in ∼5.83% of the total of transcripts compared with WT mice group (Fig. 3*C*,*G*–*I*). These differences were equally balanced between up- and downregulated genes compared with WT mice (Fig. 3*C*,*G*). All the top 10 upregulated genes in *TLR4*^−/−^ mice (by FC) at 8 weeks post-SCI were cardiac muscle related, whereas the downregulated genes were enriched in immune function (*Cd70*, *Il9r*, *Saa3*, *Ighg2c*, *Iglv1*) and ECM expression (*MMP13*; Fig. 3*H*). Furthermore, in-depth pathway analysis of all statistically significant differential genes (*TLR4* KO vs WT) at 8 weeks post-SCI revealed downstream metabolic effects (mitochondrial and ribosomal functions) and differences in neuron development pathways and synaptic function (Fig. 3*I*).

We also assessed whether differential responses between *TLR4* KO and WT mice post-SCI were time specific or whether they were consistently modulated at both subacute and chronic time points. Transcriptional changes in the injured spinal cords of *TLR4* KO compared with WT mice were apparent at both time points (1,044 and 792 differentially expressed genes at 7 d and 8 weeks post-injury, respectively), with ∼10% of these genes common at both time points. Subsequent pathway analysis revealed that ECM-related pathways were transcriptionally modulated primarily at 7 d post-SCI with some components remaining differentially expressed at 8 weeks (data not shown), whereas differences in ribosomal activity and neuronal/synaptic function were more prevalent at 8 weeks post-SCI (data not shown). Metabolic and mitochondrial responses to injury were dysregulated at both time points in *TLR4* KO mice, indicating continuous dysregulation of these pathways by TLR4 signaling over subacute to chronic SCI (data not shown).

Taken together, these results suggests that TLR4 notably plays a role in late phase biological responses to SCI. *TLR4* deletion leads to differences in ECM-related pathways at 7 d, with some components remaining differentially expressed at 8 weeks post-SCI. Furthermore, *TLR4* deletion elicits changes in cell death and cytokine pathways and in mitochondrial function and ribosomal activity that can result in differences in synaptic and neuronal function at later time points that may contribute to neuronal plasticity.

### TLR4 null mice show lower levels of chemokine and cytokine expression compared with wild-type mice at chronic stages after SCI

**Figure 4.**
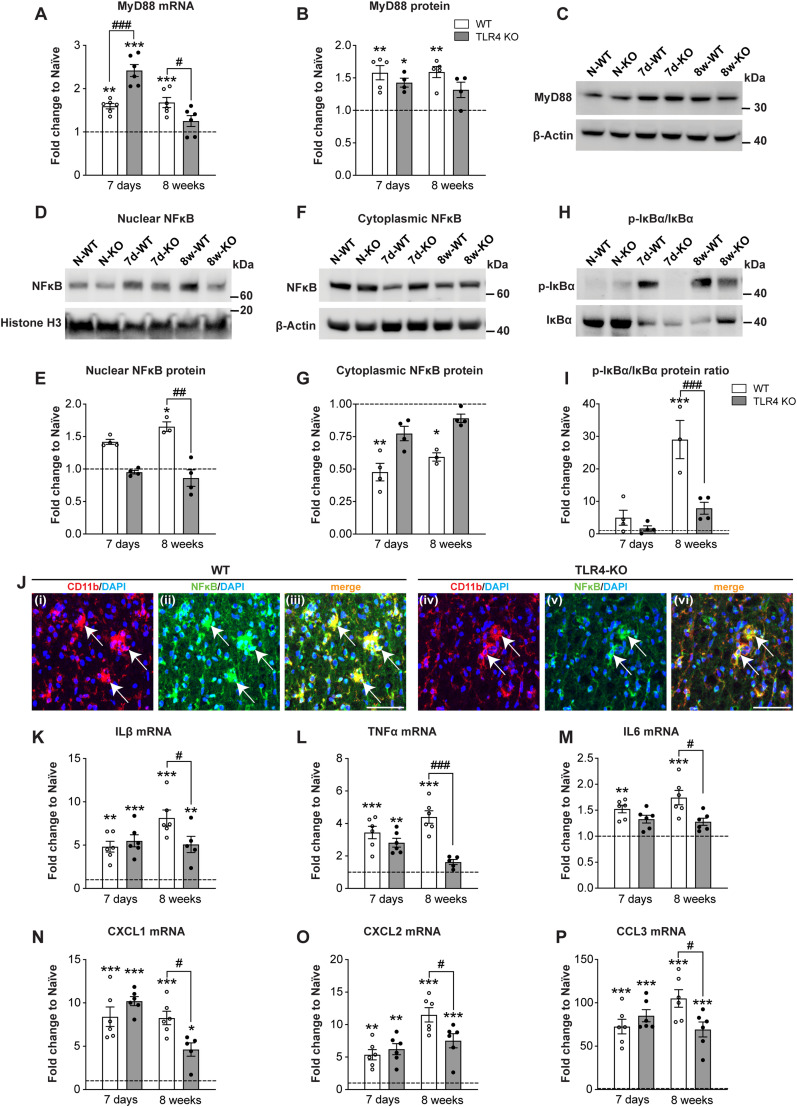
Reduced expression of MyD88, NFκB, and cytokines in chronic SCI in *TLR4* null mice. ***A–C***, Changes in *MyD88* mRNA (***A***; *n* = 6 for all groups) and protein expression (***B***,***C***; *n* = 4–5 for all groups) at 7 d and 8 weeks post-SCI in wild-type and TLR4 null mice. Note the increased expression at 8 weeks in wild-type but not *TLR4* null mice. ***D***–***I***, Western blots of nuclear (***D***,***E***) and cytoplasmic (***F***,***G***) NFκB and phospho-IκBα/IκB ratio (***H***,***I***). Note the increase at 8 weeks in nuclear NFκB (***E***) and phospho-IκBα/IκB ratio (***I***) in wild-type mice as compared with *TLR4* null mice (*n* = 3–4 for all groups). ***J***, Double immunofluorescence labeling of NFκB (green) and CD11b (red) and merge (yellow) 8 weeks after SCI in wild-type (WT; ***Ji***–***iii***) and *TLR4* KO (***Jiv***–***vi***) mice. Note strong NFκB expression in the cytoplasm and nucleus in CD11b+ macrophages in WT mice (arrows; ***Jiii***) but lack of nuclear localization in TLR4 KO mice (arrows; ***Jvi***). Also note the significant increase in expression in wild-type mice at 8 weeks compared with *TLR4* null mice in *IL-1β* (***K***), *TNFα* (***L***), *IL-6* (***M***), *CXCL1* (***N***), *CXCL2* (***O***), and *CCL3* (***P***). ***K***–***P***, *n* = 5–6 for all groups; one-way ANOVA with *post hoc* Tukey’s multiple-comparisons test; *p* < 0.0001 (***A***,***B***,***I***,***K***–***P***); *p* = 0.004 (***E***); *p* = 0.004 (***G***). *p ≤ 0.05; **p ≤ 0.01; ***p ≤ 0.001 compared with uninjured naive level. ^#^*p* ≤ 0.05; ^##^*p* ≤ 0.01; ^###^*p* ≤ 0.001 comparing the two injured genotypes. Scale bar: ***J***, 50 µm.

TLR4 like most Toll-like receptors uses the adaptor protein MyD88 to induce activation of NFκB and subsequent induction of pro-inflammatory genes including IL-1β (Medzhitov et al., 1998; Heiman et al., 2014). In addition, TLR4 can use TRIF to also activate NFκB via an MyD88-independent pathway (Yamamoto et al., 2002; Humphries et al., 2015). To further extend the RNASeq results, we assessed the expression of MyD88, NFκB activation, and several chemokines and cytokines in wild-type and *TLR4* null mice after SCI at 7 d and 8 weeks post-SCI. At the mRNA level, *MyD88* at 8 weeks post-injury was significantly higher in wild-type mice than that in *TLR4* null mice (Fig. 4*A*; one-way ANOVA; *F*_(5,30)_ = 30.45; *p *< 0.0001; with *post hoc* Tukey’s multiple-comparisons test; naive vs 7d-WT, *p *= 0.002; naive vs 7d-KO, *p *< 0.0001; naive vs 8w-WT, *p *= 0.0004; naive vs 8w-KO, *p *= ns; 7d-WT vs 7d-KO, *p *< 0.0001; 8w-WT vs 8w-KO, *p *= 0.04; *n *= 6 mice per group), suggesting a continued effect of TLR4 in modulating MyD88 at chronic phases after SCI. At the protein level, MyD88 expression after SCI was significantly higher in wild-type mice compared with uninjured controls at both survival times after injury (Fig. 4*B*,*C*; one-way ANOVA; *F*_(5,22)_ = 8.8; *p *< 0.0001; with *post hoc* Tukey’s multiple-comparisons test; naive vs 7d-WT, *p *= 0.0017; naive vs 7d-KO, *p *= 0.04; naive vs 8w-WT, *p *= 0.0014; naive vs 8w-KO, *p *= ns; 7d-WT vs 7d-KO, *p *= ns; 8w-WT vs 8w-KO, *p *= ns; *n *= 4–5 mice per group). In contrast, there was no significant increase in the injured spinal cord of TLR4 KO at 8 weeks post-SCI compared with uninjured controls (Fig. 4*B*).

Additionally, Western blot analysis showed that NFκB-P65, which is translocated to the nucleus after phosphorylation, is significantly increased in wild-type mice after SCI compared with uninjured controls and is significantly higher than *TLR4* null mice at 8 weeks post-SCI (Fig. 4*D*,*E*; one-way ANOVA; *F*_(5,15)_ = 5.581; *p *= 0.004; with *post hoc* Tukey’s multiple-comparisons test; naive vs 7d-WT, *p *= ns; naive vs 7d-KO, *p *= ns; naive vs 8w-WT, *p *= 0.04; naive vs 8w-KO, *p *= ns; 7d-WT vs 7d-KO, *p *= ns; 8w-WT vs 8w-KO, *p *= 0.007; *n *= 3–4 mice per group). The cytoplasmic p65 level in TLR4 null mice after injury remained unchanged from uninjured null mice (Fig. 4*F*,*G*; one-way ANOVA; *F*_(5,17)_ = 7.15; *p *= 0.0009; with *post hoc* Tukey’s multiple-comparisons test; naive vs 7d-WT, *p *= 0.002; naive vs 7d-KO, *p *= ns; naive vs 8w-WT, *p *= 0.03; naive vs 8w-KO, *p *= ns; 7d-WT vs 7d-KO, *p *= ns; 8w-WT vs 8w-KO, *p *= ns; *n *= 3–4 mice per group) These results were further confirmed by a significant and marked (∼30-fold) increase in phospho-IκBα/IκB ratio at 8 weeks after injury in wild-type mice as compared with *TLR4* null mice (Fig. 4*H*,*I*; one-way ANOVA; *F*_(5,15)_ = 16.71; *p *< 0.0001; with *post hoc* Tukey’s multiple-comparisons test; naive vs 7d-WT, *p *= ns; naive vs 7d-KO, *p *= ns; naive vs 8w-WT, *p *< 0.0001; naive vs 8w-KO, *p *= ns; 7d-WT vs 7d-KO, *p *= ns; 8w-WT vs 8w-KO; *p *= 0.0003; *n *= 3–4 mice per group). Reciprocal changes were also seen in levels of total IκBα (Fig. 4*H*). NFκB expression and localization assessed by immunofluorescence labeling at 8 weeks after SCI showed increased expression mainly in CD11b^+^ macrophages (Fig. 4*J*) and in some GFAP+ astrocytes but not neurons (data not shown). Wild-type mice show both nuclear and cytoplasmic labeling, whereas *TLR4* KO mice show lack of nuclear localization (Fig. 4*J*). The combined results confirm reduction of NFκB activation in the chronic period after SCI in *TLR4* null mice unlike that seen in wild-type mice.

These results were supported by findings showing significant increase in the expression of *IL-1β*, *TNFα*, *IL-6*, and chemokines (*CXCL1*, *CXCL2*, and *CCL3*) at 8 weeks after SCI in wild-type mice as compared with TLR4 null mice (Fig. 4*K–P*). These results provide evidence that the delayed expression of pro-inflammatory cytokines after SCI is reduced in *TLR4* null mice at 8 weeks post-SCI. At the earlier time point, most of these cytokines and chemokines were increased ∼10-fold with no differences between genotypes (Fig. 4*K–P*). Therefore, these results reveal an unexpected late effect of TLR4 signaling in mediating pro-inflammatory cytokine and chemokine expression in the chronic phase after SCI (Fig. 4*K*; *IL-1β*; one-way ANOVA; *F*_(5,29)_ = 18.86; *p *< 0.0001; with *post hoc* Tukey’s multiple-comparisons test; naive vs 7d-WT, *p *= 0.002; naive vs 7d-KO, *p *= 0.0004; naive vs 8w-WT, *p *< 0.0001; naive vs 8w-KO, *p *= 0.002; 7d-WT vs 7d-KO, *p *= ns; 8w-WT vs 8w-KO, *p *= 0.03; *n *= 5–6 mice per group. Figure 4*L*; *TNFα*; one-way ANOVA; *F*_(5,30)_ = 25.41; *p *< 0.0001; with *post hoc* Tukey’s multiple-comparisons test; naive vs 7d-WT, *p *< 0.0001; naive vs 7d-KO, *p *= 0.0012; naive vs 8w-WT, *p *< 0.0001; naive vs 8w-KO, *p *= ns; 7d-WT vs 7d-KO, *p *= ns; 8w-WT vs 8w-KO, *p *< 0.0001; *n *= 6 mice per group. Figure 4*M*; *IL-6*; one-way ANOVA; *F*_(5,30)_ = 9.098; *p *< 0.0001; with *post hoc* Tukey’s multiple-comparisons test; naive vs 7d-WT, *p *= 0.009; naive vs 7d-KO, *p *= ns; naive vs 8w-WT, *p *= 0.0001; naive vs 8w-KO, *p *= ns; 7d-WT vs 7d-KO, *p *= ns; 8w-WT vs 8w-KO, *p *= 0.015; *n *= 6 mice per group. Figure 4*N*; *CXCL1*; one-way ANOVA; *F*_(5,29)_ = 34.34; *p *< 0.0001; with *post hoc* Tukey’s multiple-comparisons test; naive vs 7d-WT, *p *< 0.0001; naive vs 7d-KO, *p *< 0.0001; naive vs 8w-WT, *p *< 0.0001; naive vs 8w-KO, *p *= 0.017; 7d-WT vs 7d-KO, *p *= ns; 8w-WT vs 8w-KO, *p *= 0.011; *n *= 5–6 mice per group. Figure 4*O*; *CXCL2*; one-way ANOVA; *F*_(5,30)_ = 24.59; *p *< 0.0001; with *post hoc* Tukey’s multiple-comparisons test; naive vs 7d-WT, *p *= 0.008; naive vs 7 d-KO, *p *= 0.0013; naive vs 8 weeks-WT, *p *< 0.0001; naive vs 8w-KO, *p *< 0.0001; 7d-WT vs 7d-KO, *p *= ns; 8w-WT vs 8w-KO, *p *= 0.016; *n *= 6 mice per group. Figure 4*P*; *CCL3*; one-way ANOVA; *F*_(5,30)_ = 39.15; *p *< 0.0001; with *post hoc* Tukey’s multiple-comparisons test; naive vs 7d-WT, *p *< 0.0001; naive vs 7d-KO, *p *< 0.0001; naive vs 8w-WT, *p *< 0.0001; naive vs 8w-KO, *p *< 0.0001; 7d-WT vs 7d-KO, *p *= ns; 8w-WT vs 8w-KO, *p *= 0.013; *n *= 6 mice per group).

### TLR4 null mice show differences in immune cell recruitment and phenotype early after SCI and subsequent reduction in astrogliosis and microgliosis in chronic SCI

**Figure 5.**
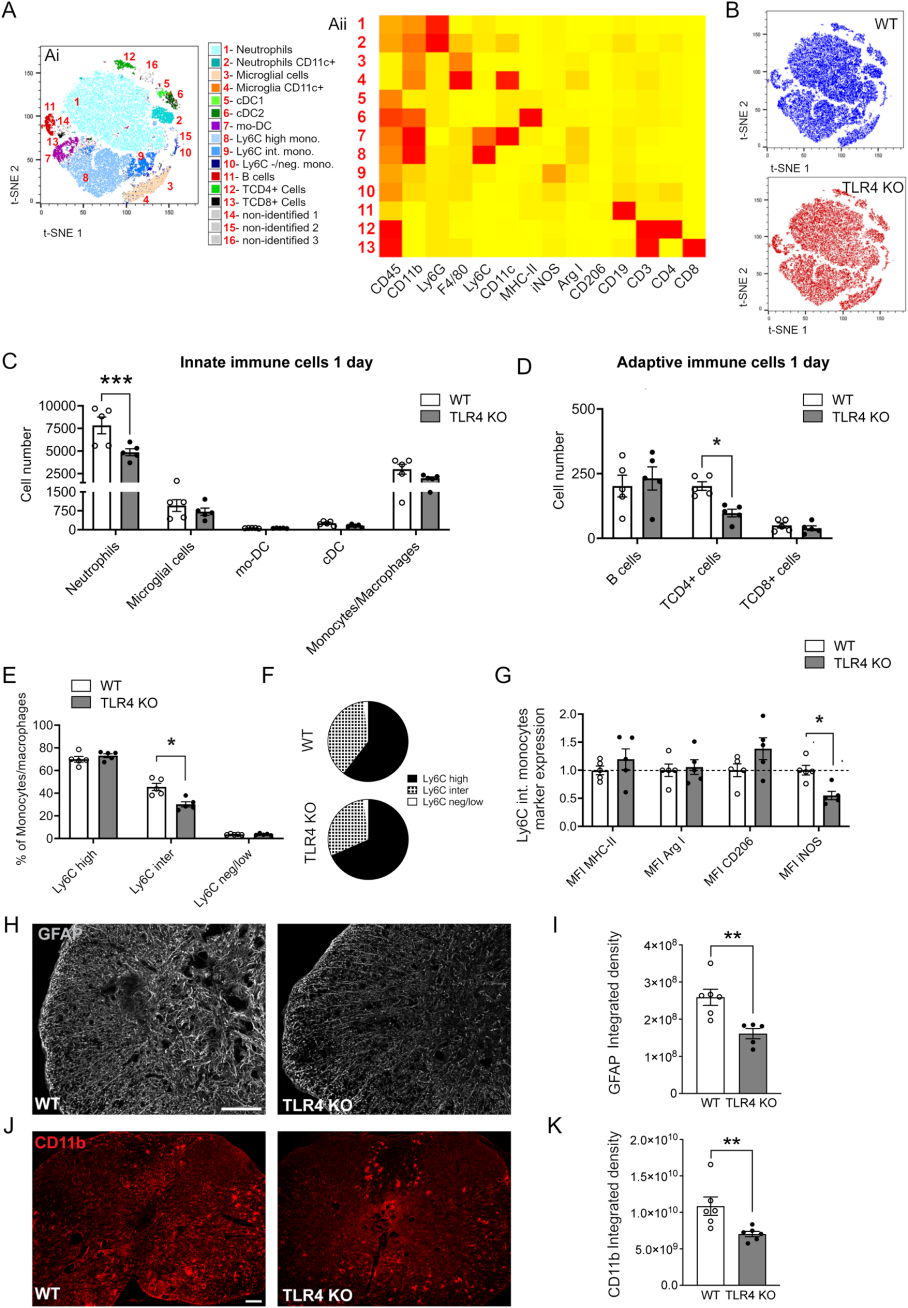
Reduced immune cell recruitment 1 d after SCI and reduced gliosis in chronic SCI in *TLR4* null mice. ***A***, (***Ai***) t-SNE flow cytometry analysis at 1 d post-SCI reveals the presence of 13 identified and 3 nonidentified CD45^+^ cell populations within the injured spinal cord. (***Aii***) Heat map showing the relative expression of extracellular markers in the 13 identified clusters. ***B***, t-SNE plot in WT and *TLR4* null mice to identify differences between them. ***C***,***D***, Graphs showing quantification of innate (***C***) and adaptive (***D***) immune cell recruitment, 1 d following spinal cord injury (results were assessed for normality using the Shapiro–Wilk test and analyzed using a two-tailed unpaired *t* test. Data are shown as mean ± SEM; *n* = 5 in KO and *n* = 5 in WT groups. ***E***, ***F***, Graphs showing the balance between Ly6C^high^, Ly6C^medium^, and Ly6C^low^ populations within WT and *TLR4* null mice 1 d after SCI. Note the reduction of Ly6C^inter^ in *TLR4* null mice compared with WT controls. ***G***, Bar graph showing changes in the expression of phenotypic markers in Ly6C^inter^ cell population. Note the significant reduction of iNOS expression in *TLR4* null mice compared with WT. ***H***–***K***, 8 weeks after SCI, there is significantly reduced immunoreactivity for GFAP (***H***,***I***) and CD11b (***J***,***K***) indicative of reduced of astrocyte and macrophage/microglial activation, respectively, in *TLR4* KO mice compared with wild-type mice (500 µm from the lesion epicenter). ***I***, *n *= 5 (*TLR4* KO) and *n *= 6 (WT); ***K***, *n *= 6 per group. Scale bar: ***H***, ***J***, 100 µm.

We designed a 15-color antibody panel for fluorescence cytometry (14 markers + L/D marker) that identified all the major leukocytes in the CNS. Single, live, CD45^+^ cells from spinal cords at 1 and 7 d post-SCI were analyzed, and samples were mapped two-dimensionally using t-SNE. Based on t-SNE data, we identified 16 phenotypically distinct clusters at 1 and 7 d (13 identified populations + 3 nonidentified; Fig. 5*Ai*; 7 d data not shown), generated a heat map showing the distinct lineage marker expression profiles for each cluster (Fig. 5*Aii*), and compared clustering analysis between both groups (WT vs *TLR4* KO mice—Fig. 5*B*). After corroborating the identification of each cluster by manual gating, we evaluated the role of TLR4 at early stages of SCI pathophysiology (1 and 7 d post-SCI). As expected, at 1 d we found a significant reduction of neutrophils and a small nonsignificant reduction in microglial and monocyte/macrophage cell numbers in *TLR4* KO mice compared with WT control (Fig. 5*C*). In line with a reduced inflammatory response in *TLR4* null mice, adaptive immune cell responses were also different in *TLR4* null mice at 1 d post-SCI, exhibiting significantly reduced CD4^+^ T-cell recruitment at the site of lesion (Fig. 5*D*). We next performed a more granular analysis to detect potential differences in phenotypic expression markers in immune cell populations between groups. We did not see differences in microglial phenotypic marker expression or cDC subpopulation balance (data no shown) but TLR4 null mice exhibited significant differences in monocyte/macrophage subpopulations. We found a significant reduction in the lymphocyte antigen 6 complex (Ly6C)-intermediate population in *TLR4* KO mice (Fig. 5*E*,*F*). This population at day 1 post-SCI showed significantly lower iNOS expression in *TLR4* null mice compared with WT mice (Fig. 5*Aii*, cluster 9, *G*). iNOS is a well-known inflammatory marker, suggesting a reduced inflammatory response in *TLR4*-deficient mice. In contrast, there was absence of changes in Ly6C high or Ly6C low monocytes/macrophages (data not shown).

At 7 d after SCI, we were unable to detect clear differences in innate or adaptive immune cell number between groups, nor in microglia, monocyte/macrophage, and cDC cell populations (data not shown), suggesting a lack of TLR4 function in late immune cell clearance after SCI. Taken together, these data indicate that TLR4 appear to play a role in the early recruitment of some immune cell types but not the later clearance of immune cells after SCI in the subacute phase at 7 d.

At 8 weeks post-SCI, we do not expect to detect much immune cell infiltration. Therefore, the late phase inflammatory responses we observed at 8 weeks (i.e., MyD88, NFκB, pro-inflammatory chemokine, and cytokine expression) are likely to be mediated by microglia and astrocytes or other cell types, which are known to express TLR4 and are capable of producing various cytokines (Heiman et al., 2014). Immunostaining for GFAP showed markedly decreased staining in TLR4 null mice compared with WT at 8 weeks post-SCI, suggesting reduced astrocyte reactivity in TLR4 KO mice in the chronic period (Fig. 5*H*,*I*; WT vs TLR4 KO, *p *= 0.004; two-tailed Mann–Whitney *U* test; *n* = 5–6 mice per group). In addition, clusters of CD11b+ macrophages/microglia observed in cross sections of the spinal cord also appeared to be greater in WT mice versus TLR4 KO mice (Fig. 5*J*,*K*; WT vs TLR4 KO, *p *= 0.004; two-tailed Mann–Whitney *U* test; *n* = 6 mice per group). There is therefore reduced astrocyte and microglial cell reactivity in TLR4 null mice compared with wild types at 8 weeks after SCI. These data suggest that TLR4 plays a role in the later activation of resident glia at chronic time points that may contribute to the chronification of the inflammatory response after SCI.

### Reduced expression of MMP9 after SCI in TLR4 null mice

**Figure 6.**
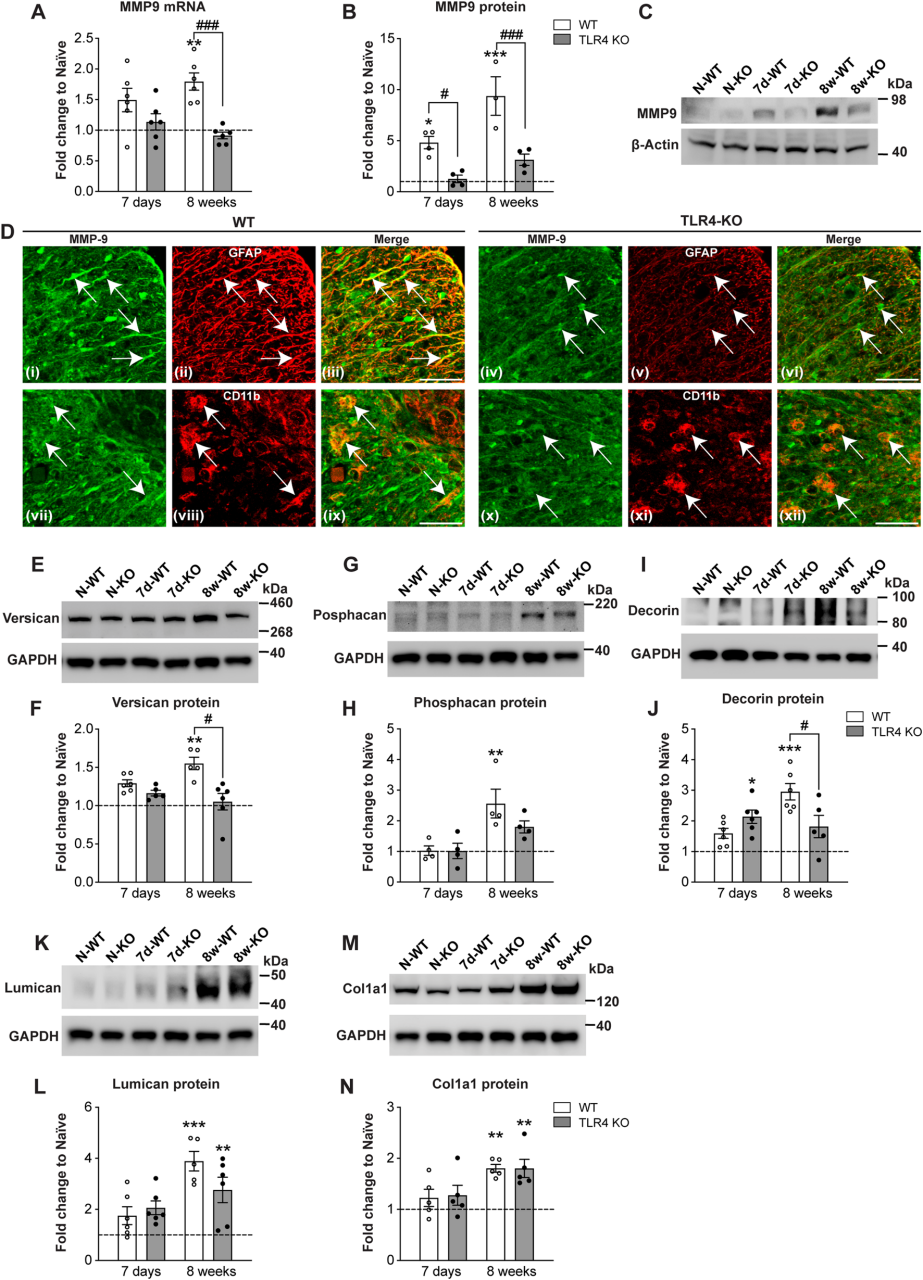
Changes in expression of MMP9 and ECM molecules. ***A***–***C***, At the mRNA level, *MMP9* expression is significantly higher in wild-type mice than that in *TLR4* KO mice at 8 weeks (***A***). A significantly higher expression of MMP9 protein was detected by Western blot in wild-type mice compared with *TLR4* null mice at 7 d and 8 weeks after SCI (***B***,***C***; ***A***, *n* = 6 for all groups; ***B***,***C***, *n* = 3–4 for all groups). ***D***, Double immunofluorescence labeling for MMP9 (green) and either GFAP (***Dii***,***v***) or CD11b (***Dviii***–***xi***; both red) and merge (yellow) at 8 weeks post-SCI. MMP9 labeling is strong in wild-type animals in GFAP^+^ astrocytes (***Diii***) and CD11b^+^ macrophages (***Dix***) but is reduced in *TLR4* null mice. Arrows indicate double labeled cells. ***E***–***N***, Changes in protein level expression detected by Western blot of various ECM molecules. Note the statistically significant increase in wild-type mice at 8 weeks in the expression of versican (***E***,***F***; *n* = 5–6 for all groups), phosphacan (***G***,***H***; *n* = 4 for all groups), and decorin (***I***,***J***; *n* = 5–6 for all groups), while *TLR4* null mice were not significantly different from uninjured controls. Expression of lumican (***K***,***L***; *n* = 5–6 for all groups) and collagen 1a1 (***M***,***N***; *n* = 5 for all groups) was increased in both genotypes at 8 weeks; one-way ANOVA with *post hoc* Tukey’s multiple-comparisons test; *p* = 0.0002 (***A***); *p* < 0.0001 (***B***,***J***,***L***); *p* = 0.0007 (***N***); *p* = 0.003 (***F***); *p* = 0.0013 (***H***). *p ≤ 0.05; **p ≤ 0.01; ***p ≤ 0.001 compared with uninjured naive level; ^#^*p* ≤ 0.05, ^##^*p* ≤ 0.01, ^###^*p* ≤ 0.001 comparing the two injured genotypes. Scale bar: ***D***, 50 µm.

The flow cytometry and RNASeq analysis led us to evaluate the expression of MMP and ECM molecules in *TLR4* KO and WT mice at subacute (7 d) and chronic (8 weeks) time points after SCI. MMPs play a key role in remodeling the ECM and in wound healing (Trivedi et al., 2019). However, excessive and continued expression of MMPs in the injured CNS can be detrimental and can lead to impairment of the blood–brain barrier, increased inflammation, neurodegeneration, and poor functional recovery (Zhang et al., 2011). Previous studies have shown that MMP9 is rapidly expressed by 1 d after SCI and diminishes by 2 weeks (Duchossoy et al., 2001; Noble et al., 2002; Goussev et al., 2003; Hansen et al., 2013). Long-term expression beyond 4 weeks has not been reported. We found that *MMP9* mRNA shows no statistically significant change at 7 d in wild-type or *TLR4* KO mice but is significantly increased at 8 weeks in wild-type mice as compared with *TLR4* KO mice, which remains at the uninjured level (Fig. 6*A*; one-way ANOVA; *F*_(5,30)_ = 7.15; *p *= 0.0002; with *post hoc* Tukey’s multiple-comparisons test; naive vs 7d-WT, *p *= ns; naive vs 7d-KO, *p *= ns; naive vs 8w-WT, *p *= 0.002; naive vs 8w-KO, *p *= ns; 7d-WT vs 7d-KO, *p *= ns; 8w-WT vs 8w-KO, *p *= 0.0004; *n *= 6 mice per group). MMP9 protein, however, is increased approximately fivefold in wild-type mice at 7 d (Fig. 6*B*,*C*), like previous reports (Hansen et al., 2013). In contrast, there is no change in MMP9 protein in *TLR4* KO mice at 7 d after injury (Fig. 6*B*). Surprisingly, at 8 weeks, MMP9 protein is increased up to ∼10-fold in wild-type mice, with no significant changes seen in *TLR4* KO mice (Fig. 6*B*,*C*; one-way ANOVA; *F*_(5,17)_ = 16.87; *p *< 0.0001; with *post hoc* Tukey’s multiple-comparisons test; naive vs 7d-WT, *p *= 0.018; naive vs 7d-KO, *p *= ns; naive vs 8w-WT, *p *< 0.0001; naive vs 8w-KO, *p *= ns; 7d-WT vs 7d-KO, *p *= 0.03; 8w-WT vs 8w-KO, *p *= 0.0004; *n *= 3–4 mice per group). Double immunofluorescence labeling shows that in wild-type mice at 8 weeks, MMP9 is expressed strongly in GFAP^+^ astrocytes and CD11b^+^ macrophages (Fig. 6*D*). This staining is markedly reduced in *TLR4* KO mice (Fig. 6*D*). The reduction in expression seen by Western blot in *TLR4* KO mice at 8 weeks therefore appears to be due to reduction in the level of expression in these glial cells. These findings indicate that TLR4 signaling can also have a late effect in remodeling ECM molecules in the chronic phase after SCI.

### Changes in expression of ECM molecules in TLR4 null mice after SCI

Several types of proteoglycans are deposited at and near the site of SCI that contribute to scar formation as well as have effects on wound healing and axon growth and sprouting (Fawcett, 2015; Tran et al., 2022). We therefore assessed the expression of several of these ECM molecules by Western blotting and immunofluorescence staining of tissue sections at 7 d and 8 weeks after SCI.

Versican and phosphacan expression detected by Western blot were significantly increased at 8 weeks after SCI in wild-type but not *TLR4* null mice (Fig. 6*E–H*). (Figure 6*F*, one-way ANOVA; *F*_(5,28)_ = 4.49; *p *= 0.003; with *post hoc* Tukey’s multiple-comparisons test; naive vs 7d-WT, *p *= ns; naive vs 7d-KO, *p *= ns; naive vs 8w-WT, *p *= 0.008; naive vs 8w-KO, *p *= ns; 7d-WT vs 7d-KO, *p *= ns; 8w-WT vs 8w-KO, *p *= 0.011; *n *= 5–6 mice per group. Figure 6*H*, one-way ANOVA; *F*_(5,18)_ = 6.44; *p *= 0.0013; with *post hoc* Tukey’s multiple-comparisons test; naive vs 7d-WT, *p *= ns; naive vs 7d-KO, *p *= ns; naive vs 8w-WT, *p *= 0.004; naive vs 8w-KO, *p *= ns; 7d-WT vs 7d-KO, *p *= ns; 8w-WT vs 8w-KO, *p *= ns; *n *= 4 mice per group.)

Decorin expression detected by Western blot was significantly increased in *TLR4* null mice at 7 d and in wild-type mice at 8 weeks (Fig. 6*I*,*J*) and being significantly higher in wild-type than that in *TLR4* null mice at 8 weeks (one-way ANOVA; *F*_(5,29)_ = 10.74; *p *< 0.0001; with *post hoc* Tukey’s multiple-comparisons test; naive vs 7d-WT, *p *= ns; naive vs 7d-KO, *p *= 0.013; naive vs 8w-WT, *p *< 0.0001; naive vs 8w-KO, *p *= ns; 7d-WT vs 7d-KO, *p *= ns; 8w-WT vs 8w-KO, *p *= 0.02; *n *= 5–6 mice per group).

Lumican expression detected by Western blot was not significantly changed at 7 d after SCI but was significantly increased between 2.5- and 4-fold in both genotypes at 8 weeks (Fig 6*K*,*L*; one-way ANOVA; *F*_(5,29)_ = 11.82; *p *< 0.0001; with *post hoc* Tukey’s multiple-comparisons test; naive vs 7d-WT, *p *= ns; naive vs 7d-KO, *p *= ns; naive vs 8w-WT, *p *< 0.0001; naive vs 8w-KO, *p *= 0.004; 7d-WT vs 7d-KO, *p *= ns; 8w-WT vs 8w-KO, *p *= ns; *n *= 5–6 mice per group).

Collagen 1a showed no difference at 7 d by Western blots but was significantly increased in both genotypes at 8 weeks, indicative of consolidation of the scar, with no differences between genotypes (Fig. 6*M*,*N*; one-way ANOVA; *F*_(5,24)_ = 6.3; *p *= 0.0007; with *post hoc* Tukey’s multiple-comparisons test; naive vs 7d-WT, *p *= ns; naive vs 7d-KO, *p *= ns; naive vs 8w-WT, *p *= 0.008; naive vs 8w-KO, *p *= 0.008; 7d-WT vs 7d-KO, *p *= ns; 8w-WT vs 8w-KO, *p *= ns; *n *= 5 mice per group).

**Figure 7.**
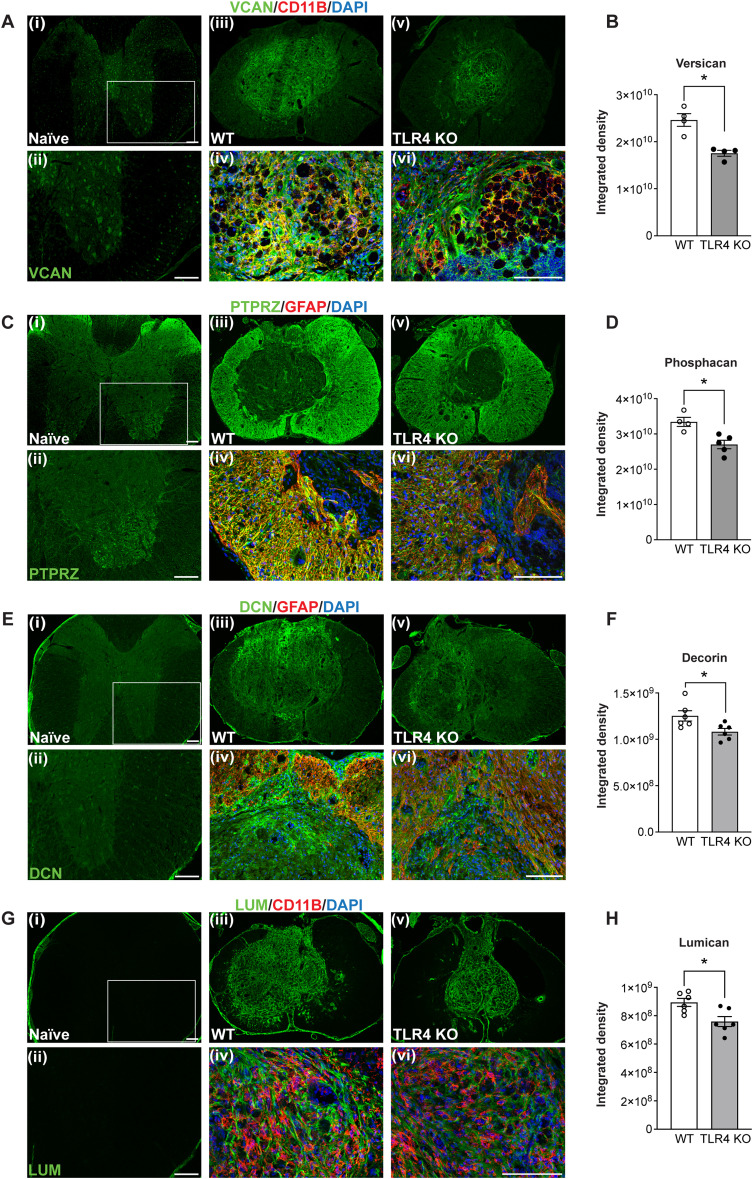
Immunostaining showing localization of ECM molecules 8 weeks after SCI. ***A***, ***B***, Versican immunostaining. In the uninjured spinal cord, versican is detected at very low levels in discrete cells in the white and gray matter by immunofluorescence (***Ai***,***ii***). After injury, versican expression is increased in wild-type and *TLR4* null mice in the central core of the lesion where it is localized to CD11b^+^ macrophages (***Aiii***–***vi***). Quantification shows significantly lower expression of versican in *TLR4* null mice compared with wild-type mice (***B***; *n* = 4 for all groups). ***C***, ***D***, Phosphacan immunolabeling is detected at low levels in the uninjured spinal cord in the dorsal and ventral gray matter, which at higher magnification (***Cii***) appears as punctate staining surrounding neurons. After SCI, phosphacan expression is markedly increased in astrocytes in the white matter with strong labeling along the lesion border in both genotypes (***Ciii***–***vi***). Note the expression level is lower in *TLR4* null mice compared with wild-type mice (***D***; *n* = 4–5 for all groups). ***E***, ***F***, Decorin staining. Immunostaining for decorin showed very weak labeling in the uninjured spinal cord (***Ei***,***ii***). After SCI, decorin staining in both genotypes is markedly increased but confined mainly to the central core of the lesion and in CNS tissue immediately dorsal (***Eiii***–***vi***). Some decorin staining colocalized with GFAP staining (***Civ***) but the majority of the staining is within the GFAP-negative central core of the lesion (***Eiii***–***vi***). Quantification shows a small but significant reduction in *TLR4* null mice (***F***; *n* = 6 for all groups). ***G***, ***H***, Lumican labeling. Lumican is not detectable in the uninjured spinal cord (***Gi***,***ii***). Its expression increases after SCI and is localized to the same region as decorin. It does not show colocalization with CD11b^+^ macrophages (***Giv***,***vi***) but appears to be in the matrix surround the cells. Lumican staining is also significantly lower in *TLR4* null mice compared with wild-type mice (***H***; *n* = 6 for all groups), *p ≤ 0.05. Two-tailed Mann–Whitney *U* test (***B***,***D***,***F***,***H***); *p *= 0.02 (***B***); *p *= 0.015 (***D***); *p *= 0.04 (***F***); *p *= 0.04 (***H***). Scale bar: for all panels, 100 µm.

To confirm Western blot findings and assess spatial expression, we next performed immunofluorescence analysis of several of these ECM molecules in tissue sections at 8 weeks post-SCI. Versican was not well detected by immunofluorescence in the uninjured spinal cord (Fig. 7*Ai*,*ii*), but 8 weeks after injury expression was strongly detected in wild-type mice in the central core of the lesion in the area that contains non-neuronal cells (Fig. 7*Aiii*,*iv*). This staining was diminished in *TLR4* null mice (Fig. 7*Av*,*vi*,*B*; WT vs KO, *p *= 0.02; two-tailed Mann–Whitney *U* test; *n *= 4 mice per group). Phosphacan labeling was detected by immunofluorescence in the uninjured spinal cord in discrete regions of the dorsal and ventral gray matter. This appeared as punctate staining especially surrounding ventral motor neurons (Fig. 7*Ci*,*ii*). Its expression was markedly increased in wild-type mice in astrocytes along the lesion border and in white matter astrocytes at 8 weeks after SCI (Fig. 7*Ciii*,*iv*). In line with the Western blot results, staining for phosphacan is significantly reduced in *TLR4* KO mice at 8 weeks (Fig. 7*Cv*,*vi*,*D*; WT vs KO, *p *= 0.015; two-tailed Mann–Whitney *U* test; *n *= 4–5 mice per group).

In the uninjured spinal cord, decorin showed very weak labeling of astrocytes (Fig. 7*Ei*,*ii*) After SCI, immunostaining in wild-type mice was localized mainly to the central core of the lesion that contains non-neuronal cells (Fig. 7*Eiii*,*iv*). This staining is reduced in *TLR4* null mice (Fig. 7*Ev*,*vi*,*F*; WT vs *TLR4* KO, *p *= 0.04; two-tailed Mann–Whitney *U* test; *n *= 6 mice per group).

No immunostaining was detected for lumican in the uninjured spinal cord (Fig. 7*Gi*,*ii*). Immunostaining was markedly increased at 8 weeks post-SCI throughout the central core of the lesion in wild-type mice (Fig. 7*Giii*,*iv*) and was significantly lower in *TLR4* null mice (Fig. 7*Gv*,*vi*,*H*; WT vs KO, *p *= 0.04; two-tailed Mann–Whitney *U* test; *n *= 6 mice per group). The staining pattern for lumican appeared like that of decorin, showing labeling along the lesion border as well as of cells within the lesion core.

**Figure 8.**
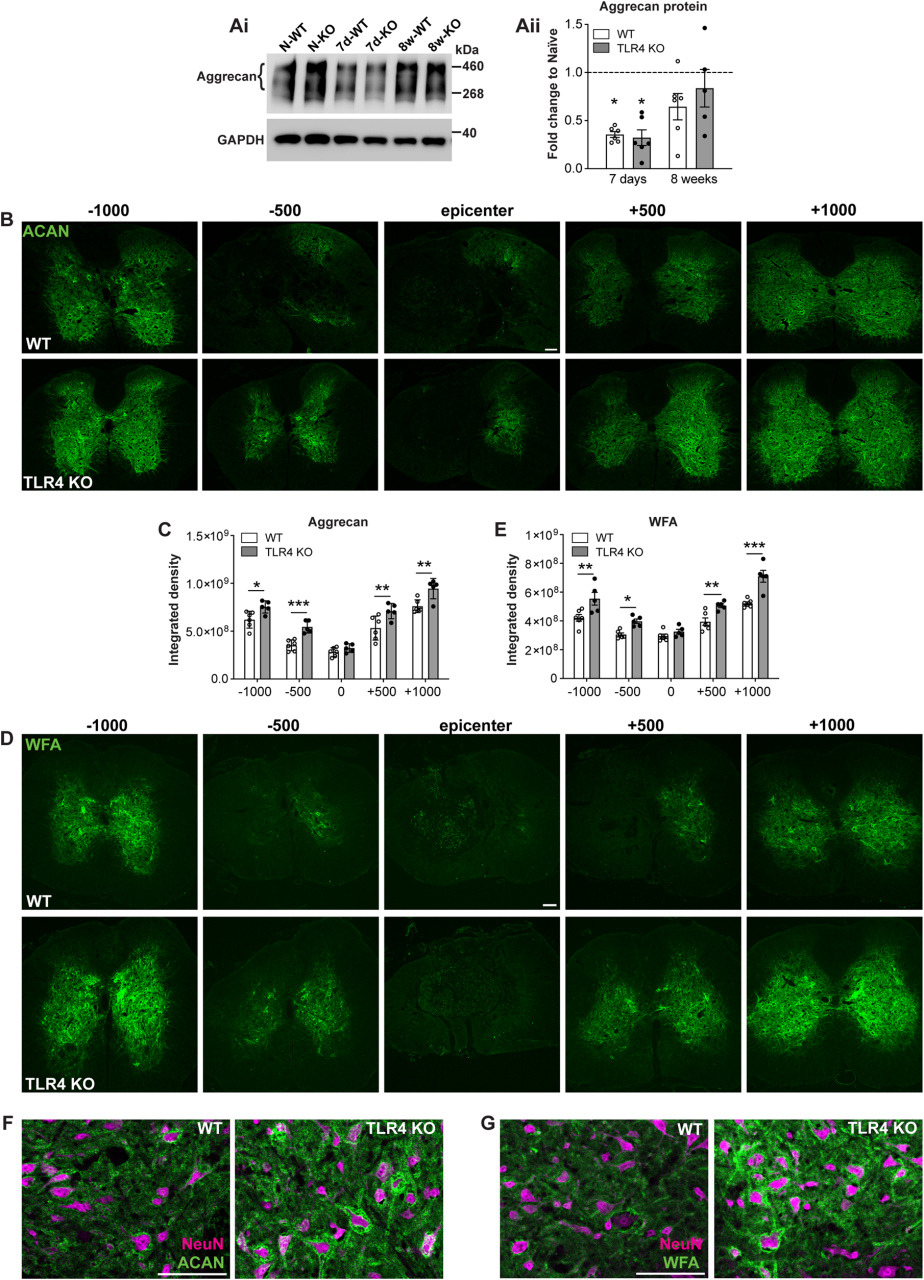
Expression of aggrecan and localization of WFA staining. ***Ai***, Western blot analysis of aggrecan in *TLR4* null and wild-type mice at 7 d and 8 weeks post-SCI. ***Aii***, Quantification of Western blots. Note that expression is significantly reduced at 7 d in both genotypes as compared with uninjured levels (*n* = 5–6 for all groups). ***B***, Immunofluorescence staining of aggrecan at 8 weeks after SCI shows loss of expression at and near the lesion epicenter in both groups. ***C***, Quantification shows significantly greater expression of aggrecan on either side of the lesion epicenter in *TRL4* null mice compared with wild-type mice (*n* = 5–6 for all groups). ***D***, WFA staining shows similarities to aggrecan staining with more intense labeling in *TLR4* null mice compared with wild-type mice, which is confirmed by quantification (***E***; *n* = 5–6 for all groups). ***F***, ***G***, Double labeling for aggrecan and NeuN (***F***) and WFA and NeuN (***G***) shows that aggrecan and WFA labeling is localized to regions surrounding NeuN^+^ neurons, a pattern that resembles PNNs. *n* = 5–6 for all groups; *p ≤ 0.05; **p ≤ 0.01; ***p ≤ 0.001. ***Aii***, One-way ANOVA with *post hoc* Tukey’s multiple-comparisons test, *p* = 0.006; (***C***,***E***), two-way RM-ANOVA; genotype effect, with *post hoc* Bonferroni’s multiple-comparisons test; *p *= 0.0016 (***C***); *p *< 0.0001 (***E***). Scale bar: for all panels, 100 µm.

Aggrecan (ACAN) expression detected by Western blots was significantly reduced at 7 d in both genotypes compared with uninjured levels with no differences observed between genotypes (Fig. 8*Ai*,*ii*). At 8 weeks post-SCI, the levels were not significantly different from uninjured levels but the level in *TRL4* null mice appeared to be somewhat higher than WT (but not reaching significance; Fig. 8*Aii*; one-way ANOVA; *F*_(5,29)_ = 4.12; *p *= 0.006; with *post hoc* Tukey’s multiple-comparisons test; naive vs 7d-WT, *p *= 0.04; naive vs 7d-KO, *p *= 0.03; naive vs 8w-WT, *p *= ns; naive vs 8w-KO, *p *= ns; 7d-WT vs 7d-KO, *p *= ns; 8w-WT vs 8w-KO, *p *= ns; *n *= 5–6 mice per group). The slightly lower levels of aggrecan at 8 weeks post-SCI in WT mice after SCI agree with our previous report (Didangelos et al., 2016). Immunofluorescence labeling in serial cross sections showed that aggrecan is localized to the gray matter and reduced at and near the lesion epicenter in both genotypes (Fig. 8*C*). Quantification of this labeling showed a significantly higher aggrecan expression in *TLR4* null mice at 8 weeks after SCI on either side of the lesion epicenter (Fig. 8*B*,*C*; two-way RM-ANOVA; genotype effect, *F*_(1,9)_ = 19.89; *p *= 0.0016; with *post hoc* Bonferroni’s multiple-comparisons test; *p*_(−1000)_ = 0.03; *p*_(−500)_ = 0.001; *p*_(epicenter)_ = ns; *p*_(+500)_ = 0.002; *p*_(+1000)_ = 0.0017; *n *= 5–6 mice per group). At higher magnification, aggrecan labeling appears to surround NeuN+ neuronal cell bodies (Fig. 8*F*), similar to that reported for perineuronal nets (PNNs) (Takiguchi et al., 2021) with increased aggrecan labeling of PNNs apparent in *TLR4* null mice.

The lectin, WFA, is also known to be localized to PNNs (Takiguchi et al., 2021) and was found to surround NeuN+ neurons and label more strongly in *TLR4* null mice than that in wild-type mice at 8 weeks after SCI (Fig. 8*D*,*E*,*G*). The WFA staining was similar to that of aggrecan, in that it surrounded NeuN^+^ neurons (Fig. 8*G*) and like aggrecan, the WFA staining at 8 weeks is significantly greater in *TLR4* null mice rostral and caudal to the lesion epicenter as compared with wild-type mice (Fig. 8*E*; two-way RM-ANOVA; genotype effect *F*_(1,9)_ = 60.24; *p *< 0.0001; with *post hoc* Bonferroni’s multiple-comparisons test; *p*_(−1000)_ = 0.0011; *p*_(−500)_ = 0.03; *p*_(epicenter)_ = ns; *p*_(+500)_ = 0.007; *p*_(+1000)_ = *p *< 0.0001; *n *= 5–6 mice per group). PNNs are thought to stabilize synapses located on the neuronal cell bodies and proximal dendrites and increase during maturation of the nervous system (Takiguchi et al., 2021). The increased expression of WFA and aggrecan in the injured spinal cord of *TLR4* KO mice suggest that in the chronic phase (8 weeks after injury), better consolidation and stabilization of synapses could contribute to improved functional recovery.

**Figure 9.**
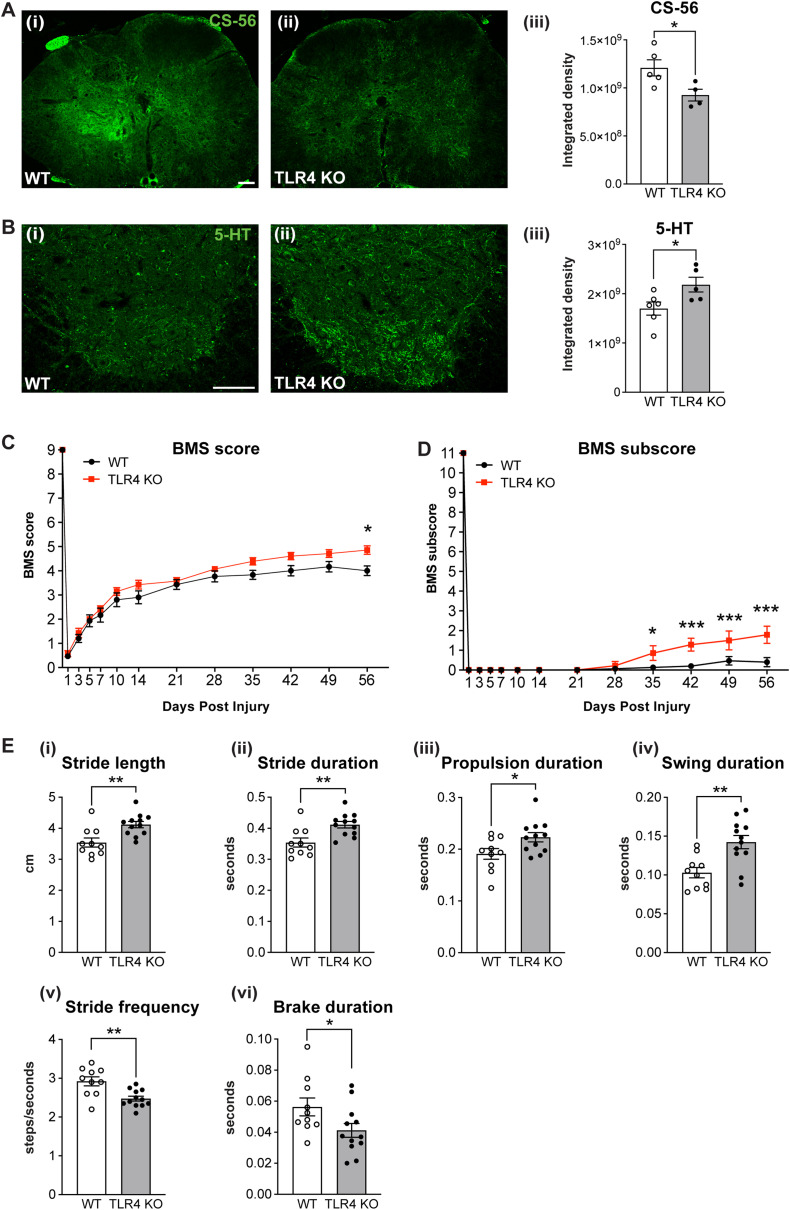
Reduction in CSPG GAG staining, increase in 5-HT sprouting, and improvement in locomotor recovery in *TLR4* null mice 8 weeks after SCI. ***Ai***, ***ii***, CS-56 monoclonal antibody labeling of CSPG GAG chains labels wild-type injured spinal cord at 8 weeks more strongly than *TLR4* null mice. Images taken at the same distance from the lesion epicenter (500 µm). ***Aiii***, Quantification shows significant reduction in CS-56 labeling in *TLR4* null mice compared with wild-type mice (*n* = 4–6 per all group). ***Bi***, ***ii***, 5-HT immunoreactivity in the ventral horn region 1 mm caudal to the lesion epicenter shows increased innervation in *TLR4* null mice (***Bii***) compared with wild-type mice (***Bi***). Quantification shows significant increase in 5-HT labeling in *TLR4* null mice compared with wild-type mice (***Biii***; *n* = 5–6 per all group). ***C***, Locomotor recovery assessed by BMS analysis shows delayed improvement in locomotor recovery in *TLR4* null mice by 8 weeks; *n *= 14 (*TLR4* KO) and *n *= 15 (WT). Two-way RM-ANOVA; time × genotype effect with *post hoc* Bonferroni’s multiple-comparisons test; *p *= 0.02 (***D***). BMS subscores, which evaluate finer aspects of locomotor control, show significant improvement starting from 35 d post-SCI; *n *= 14 (*TLR4* KO) and *n *= 15 (WT). Two-way RM-ANOVA; time × genotype effect with *post hoc* Bonferroni’s multiple-comparisons test; *p *< 0.0001. ***E***, DigiGait analysis shows significant improvement is several parameters of gait in *TLR4* null mice compared with wild-type mice: (i) stride length, *p *= 0.005; (ii) stride duration, *p *= 0.005; (iii) propulsion duration, *p *= 0.04; (iv) swing duration, *p *= 0.003; (v) stride length, *p *= 0.005; (vi) brake duration, *p *= 0.04; two-tailed Mann–Whitney *U* test; *n *= 12 (*TLR4* KO) and *n *= 10 (WT); *p ≤ 0.05; **p ≤ 0.01. Scale bar: ***A***, ***B***, 100 µm.

The glycosaminoglycan (GAG) component of recognized by the CS-56 monoclonal antibody have been shown to reduce axon growth after SCI (McKeon et al., 1991; Bradbury and Carter, 2011; Hussein et al., 2020; Fawcett and Kwok, 2022). Immunofluorescence staining for the CS-56 antibody showed increased labeling in wild-type mice 500 µm adjacent to the lesion epicenter at 8 weeks after SCI, which was significantly reduced in *TLR4* null mice (Fig. 9*Ai*–*iii*; two-tailed Mann–Whitney *U* test; *n *= 4–6 mice per group; *p *= 0.03). CS-56 labeling was barely detectable at 1 mm caudal to the lesion epicenter in both groups (data not shown). To assess the possible effects of this on neuronal plasticity after SCI, we examined the innervation of serotonergic fibers in the ventral horn caudal to the lesion. These descending 5-HT fibers from neurons in the raphe nuclei are important for locomotor recovery. We detected a significant increase in 5-HT innervation of the ventral horn 1 mm caudal to the lesion in *TLR4* null mice as compared with wild-type mice (Fig. 9*Bi*–*iii*; two-tailed Mann–Whitney *U* test; *n *= 5–6 mice per group; *p *= 0.03). This could suggest that reduction in scar-associated CSPGs in *TLR4* null mice at 500 µm could permit increased sprouting of 5-HT innervation more caudally at 1 mm. These findings are supported by our RNASeq data showing that TLR4 could play a role in synaptic and neuronal functions in the chronic phase after SCI.

### TLR4 null mice show improved recovery of locomotor function at late post-injury stages

We next assessed if the differences in inflammation, cell death, and ECM expression had any effects on locomotor recovery over an 8 week period after SCI. Locomotor recovery was assessed using the BMS and DigiGait analysis. The BMS analysis, which evaluates various aspects of hindlimb movement, foot placement, weight-bearing, balance, trunk stability, and coordination in freely moving mice using a 9-point scale (Basso et al., 2006), showed a small but significant improvement in locomotor recovery in the *TLR4* KO group at 8 weeks post-SCI as compared with wild-type SCI controls (Fig. 9*C*; two-way RM-ANOVA; time × genotype effect, *F*_(12,324)_ = 2.03; *p *= 0.02; with *post hoc* Bonferroni’s multiple-comparisons test; *p*_(56)_ = 0.016; *n *= 14–15 mice per group). Additionally, the BMS subscores, which evaluate finer aspects of locomotor control, showed significant functional improvement in *TLR4* KO mice starting from day 35 onward (Fig. 9*D*; two-way RM-ANOVA; time × genotype effect, *F*_(12,324)_ = 4.98; *p *< 0.0001; with *post hoc* Bonferroni’s multiple-comparisons test; *p*_(35)_ = 0.04; *p*_(42)_ = 0.0002; *p*_(49)_ = 0.0005; *p*_(56)_ < 0.0001; *n *= 14–15 mice per group). Furthermore, several gait parameters assessed by DigiGait analysis showed improvements in *TLR4* KO mice as compared with wild-type mice at 8 weeks post-SCI (Fig. 9*E*). This analysis showed significant increases in stride length, and stride duration, as well as swing phase and propulsion phase. *TLR4* null mice also showed a reduction in stride frequency and brake duration, both indicators of improved locomotor control (Fig. 9*E*; WT vs KO, (i) stride length, *p *= 0.005; (ii) stride duration, *p *= 0.005; (iii) propulsion duration, *p *= 0.04; (iv) swing duration, *p *= 0.003; (v) stride length, *p *= 0.005; (vi) brake duration, *p *= 0.04; two-tailed Mann–Whitney *U* test; *n* = 10–12 mice per group). Although the improvement in BMS is small, this test evaluates control of multiple joints and axial regions, so a small improvement in BMS is still an important indicator when coupled with improvement in six measures in the DigiGait analysis, an independent measure of locomotor control. Interestingly, the improvements detected with the BMS and DigiGait are seen in the chronic phase after SCI, suggesting that late occurring changes in *TLR4*-mediated responses that affect inflammation and tissue pathology may contribute to such late recovery.

## Discussion

**Figure 10.**
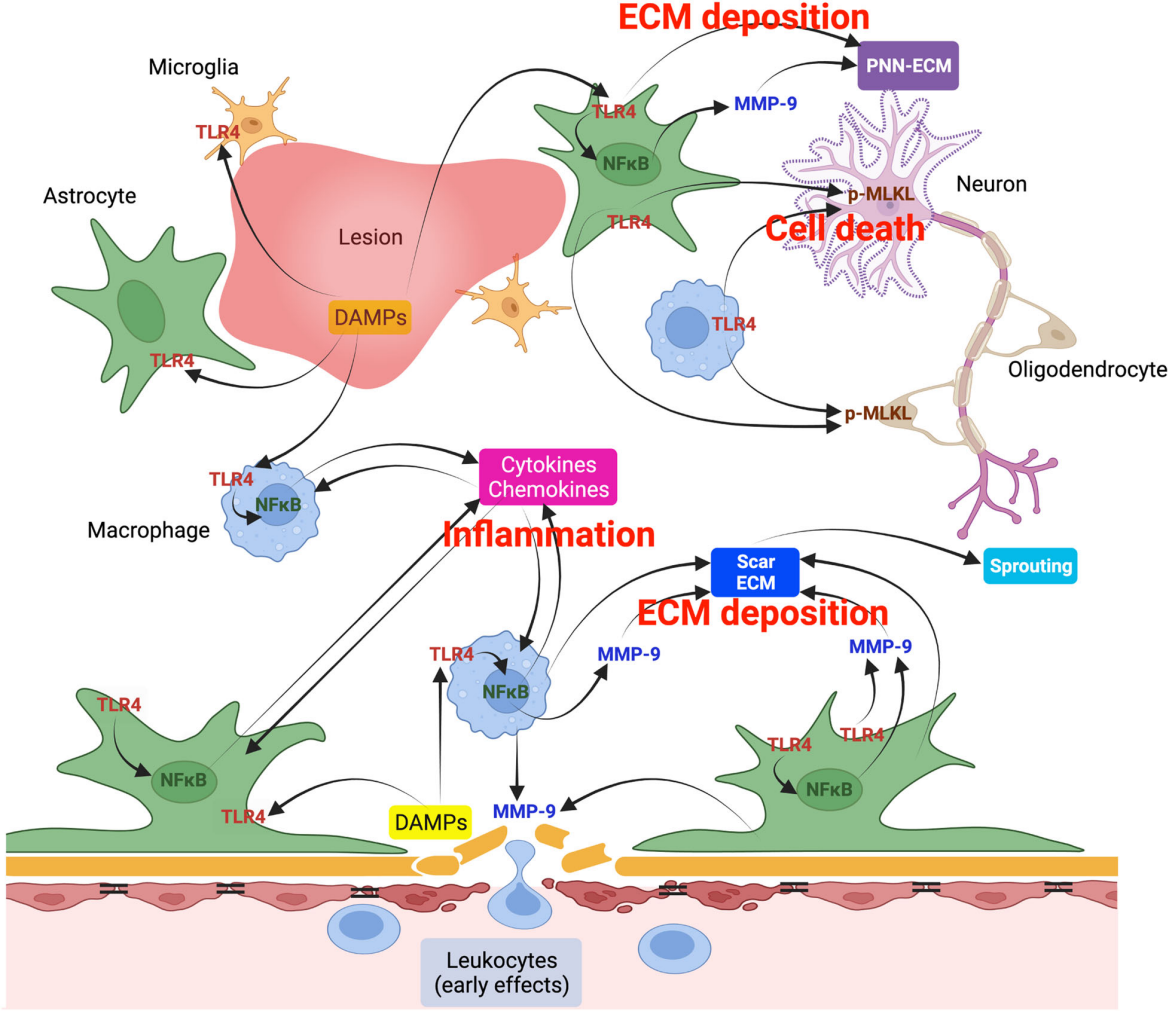
Schematic diagram illustrating the key findings of the role of TLR4 signaling after SCI. Except for the bottom of the figure which shows the early effects of TLR4 in the infiltration of leukocytes, the rest of the diagram focuses mainly on the chronic effects of TLR4, 8 weeks after SCI. TLR4 is expressed in astrocytes and macrophages at 7 d and in astrocytes at 8 weeks. It regulates NFκB signaling in macrophages and astrocytes and possibly downstream effects on chemokine and cytokines expression. TLR4 also regulates expression and deposition of PNN- and scar-related ECM molecules. At the chronic stage, TLR4 signaling via non-neuronal cells also appears to regulate the necroptosis (cell death) pathway (p-MLKL) in neurons and possibly also oligodendrocytes (data not shown). TLR4 and NFκB can also influence expression of MMP9 that may play a role in modulating the deposition of PNN- and scar-related ECM molecules in divergent ways. Three key components of the TLR4 response in chronic SCI (cell death, inflammation, and ECM deposition) are depicted in red. The reduction in necroptosis, inflammatory regulators, and modulation of scar- and PNN-related ECM molecules observed in *TLR4* null mice contribute in varying degrees to improved sprouting of 5-HT axons and improvement in locomotor recovery during the chronic period after SCI.

We show that TLR4 signaling has unexpected late effects in modulating molecular, cellular, and extracellular matrix changes in response to CNS injury. At a chronic stage after SCI (8 weeks post-injury), when inflammatory and secondary degenerative processes are typically considered to be complete or to have reached a plateau (Donnelly and Popovich, 2008; David and Lopez-Vales, 2021), mice with a targeted deletion of *TLR4* show reduced myelin and neuronal loss, and decreased expression of pro-inflammatory chemokines and cytokines compared with wild-type mice, which correspond with modest improvements in locomotor recovery at late stages post-injury. Interestingly, *TLR4* deletion also prevented late activation of the necroptosis pathway and of NFκB that was observed in wild-type mice at this chronic time point. These changes may reflect a chronic response to early post-injury changes, such as the reduction in neutrophil and CD4^+^ T-cell infiltration, and reduction of Ly6C^inter^ cells expressing iNOS that were detected in *TLR4* null versus wild-type mice 1 d after injury. Late-stage effects of *TLR4* deletion were also evident by decreased glial activation and reduced deposition of several alarmin-associated ECM molecules during the chronic period, which likely contribute to the reduction of chemokine and cytokine expression in *TLR4* null mice at this time point. On the other hand, the deposition of matrix molecules involved with PNNs were found to be increased in the ventral horn of *TLR4* null mice compared with wild-type mice at 8 weeks, suggesting better consolidation of synapses in *TLR4* null mice and indicating that TLR4 plays a modulatory role in the balance of plasticity-promoting (beneficial) and alarmin-promoting (detrimental) ECM bioavailability in the chronic injury environment. The reduction of CSPGs (CS-56 labeling) near the injury site in *TLR4* null mice was associated with greater serotonergic innervation of motor neurons in the ventral horn caudal to the lesion. These changes may contribute to the improvement in locomotor recovery in *TLR4* null mice. These results point to late effects of TLR4 signaling after CNS injury that mediate chronic responses that have an impact on tissue repair and functional recovery. These results are summarized in Figure 10.

The improved function in *TLR4* null mice at late stages after SCI may be due to a variety of factors. These include the reduced loss of ventral horn neurons in *TLR4* null mice. Our work suggests decreased necroptosis after SCI in *TLR4* null mice. This could be due to the indirect effects of TLR4 signaling in non-neuronal cells triggering activation of RIPK3 and MLKL in neurons (He et al., 2011). RIPK1–RIPK3 heterodimers induce the recruitment of MLKL that triggers necroptosis. Phosphorylated MLKL is translocated to the plasma membrane where it compromises membrane integrity, causing cell death (Humphries et al., 2015). The kinase activity of RIP3 is essential for necrotic cell death as RIPK3-deficient mice are resistant to TNF-induced necrosis (He et al., 2009). In our work, we see reduced phosphorylation of RIPK3 and MLKL in *TLR4* null mice at 8 weeks after injury. Furthermore, we also see reduction in TNF in *TLR4* null mice at 8 weeks that would also contribute to reduced necroptosis (Humphries et al., 2015). TLR signaling trigger signal transduction cascades causing activation of transcription factors such as NFκB and induction of pro-inflammatory genes including *TNF* and *IL-1β* (Heiman et al., 2014; Humphries et al., 2015), both of which are reduced in *TLR4* null mice compared with wild-type mice at 8 weeks post-injury. TLR4 can also activate NFκB and thus induce expression of other inflammatory genes in non-neuronal cells. In addition to IL-6, we also detected a reduction in several chemokines (*CXCL1*, *CXCL2*, and *CCL3*). These changes in cell death and cytokine pathways could lead to the loss of neurons, oligodendrocytes, and indirectly to myelin loss that can collectively contribute to loss of function after SCI. The relatively modest functional effects of *TLR4* deletion that we observed aligns with the concept that manipulation of only one target is not sufficient to elicit substantial recovery after SCI due to the multitude of factors that contribute to overall loss of function after SCI (Griffin and Bradke, 2020). Nevertheless, the identification of individual targets is important, particularly those that mediate multiple secondary injury inflammatory processes, as we demonstrate here for TLR4.

Flow cytometry analysis showed significant reduction in certain populations of infiltrating immune cells (neutrophil, CD4^+^ T-cells, inflammatory Ly6C^int^ monocyte/macrophages that express iNOS) early at 1 d after SCI. This was followed at 8 weeks with significant reduction in activation of macrophage/microglial and astrocytes, suggesting that the late reduction in chemokine and cytokine expression in *TLR4* null mice may be due to the effects of TLR4 signaling in resident glia, all of which express TLRs (Owens, 2009; Heiman et al., 2014). Unlike astrocytes, the role of TLRs in macrophages and microglia are well characterized (Heiman et al., 2014; Li et al., 2021). There is evidence, however, that TLR2 and TLR4 signaling is associated with the release of IL-1β, TNFα, IL-6, and various chemokines and cytokines, which we show are reduced in *TLR4* null mice after SCI. Some of the effects on astrocytes and microglia could also be mediated via reciprocal interactions between these cells as suggested previously (Owens, 2009). Furthermore, we show that TLR4 expression is increased in astrocytes and CD11b^+^ macrophages/microglia in the injured spinal cord. Stimulation of other TLRs such as TLR3 in human astrocytes can induce expression of anti-inflammatory cytokines and neuroprotective factors (Li et al., 2021). Thus, different TLRs can exert different effects. Sex differences have also been noted in the effects of TLR4 deficiency having detrimental effects in males after SCI, using a naturally occurring *TLR4* mutant mouse (Kigerl et al., 2007; Impellizzeri et al., 2015; Church et al., 2016). There is evidence that TLR4 expression in macrophages is higher in males (Klein and Flanagan, 2016). More work is needed on male mice to determine if such chronic effects of TLR4 that we see in female mice are also detected in male mice after SCI or whether the chronic effects of TLR4 differ between the sexes. In other work, inactivation of TLR4 with microRNA-940 improved recovery after SCI (Wang et al., 2019). In the current study, we used female mice with a targeted deletion of *TLR4* and find significant improvement in various parameters in the chronic period after SCI.

There is little reported about the influence of TLR4 on neutrophils in SCI. Studies in ischemic stroke indicate that neutrophils that lack TLR4 produce less reactive oxygen species, have more phagocytic activity, and are preferentially engulfed by microglia (Duran-Laforet et al., 2021). Our flow cytometry results showed that lack of TLR4 reduces the number of neutrophils in the spinal cord 1 d after SCI. TLR4 is known to be expressed on the surface of immune cells and glia including neutrophils and on microglia, astrocytes, and oligodendrocytes (Kigerl et al., 2007; Zivkovic et al., 2021). Chemokines and cytokines produced by microglia and astrocytes also influence neutrophil recruitment to the site of injury (Pineau and Lacroix, 2007; Alizadeh et al., 2019; Pelisch et al., 2020; Zivkovic et al., 2021). Early activation of TLR4 on neutrophils can induce generation of reactive oxygen species and cytokine expression (Zivkovic et al., 2021) and contribute to secondary damage in SCI. Recruitment of neutrophils is also regulated directly or indirectly via TLR4 expressed on endothelial cells that promotes leukocyte rolling and adhesion to microvessels (Zhou et al., 2009). We also observed a reduction in monocyte recruitment and iNOS expression after SCI in *TLR4* null mice. Additionally, the recruitment of CD4^+^ T-cells is reduced in *TLR4* KO after SCI and needs further detailed analysis. TLR4 activation also increases permeability of the blood–brain barrier and thus increases neutrophil and other immune cell recruitment (Hollidge et al., 2021). TLR4 may therefore act via a variety of direct and indirect mechanisms to promote neutrophil and immune cell entry into the injured spinal cord.

We recently reported that CSPGs play a critical role in preventing the resolution of inflammation after SCI by blocking the conversion of pro-inflammatory macrophages to a pro-repair phenotype (Francos-Quijorna et al., 2022). We and others have also shown that failure of such conversion of macrophage and microglial phenotype underlies some of the detrimental effects seen after SCI (Kigerl et al., 2009; Kroner et al., 2014; Francos-Quijorna et al., 2022). We reported that enzymatic digestion of CSPG GAGs with chondroitinase ABC (Ch ABC) reduces pro-inflammatory responses in vivo (Francos-Quijorna et al., 2022). Importantly, we also showed that the CSPG-induced induction of a pro-inflammatory phenotype in pro-repair macrophages in vitro is dependent on TLR4 (Francos-Quijorna et al., 2022), indicating a connection between CSPGs and TLR4 in driving detrimental pro-inflammatory responses after SCI. In support of this hypothesis, our current in vivo data demonstrated reduced recruitment of neutrophils and CD4^+^ T-cells and iNOS expressing Ly6C^int^ monocytes/macrophages in *TLR4* null mice early after injury and a subsequent reduced expression of inflammatory chemokines and cytokines in *TLR4* null mice in the chronic phase of SCI. Together these data highlight an important role for TLR4 signaling in amplifying and extending chronic inflammatory pathology in traumatic CNS injuries. *Tlr4* was reported to be expressed in macrophage/microglia and astrocytes after SCI (Kigerl et al., 2007). We now show that TLR4 protein is expressed in macrophages early after injury (7 d) while astrocytes express TLR4 during both the subacute (7 d) and chronic (8 weeks) periods. TLR4 signaling in these glia is expected to mediate some of their early responses to injury, including expression of chemokines/cytokines (Heiman et al., 2014). We show here that TLR4 can also have delayed effects during the chronic period after SCI that influences cytokine expression, scar formation, secondary damage, and functional recovery. We do not know whether these delayed effects are independent of the early changes. Future studies could evaluate conditional knock-out of *TLR4* in specific cell types either at early or late stages post-injury, to further determine the contribution of TLR4 signaling in non-neuronal cells to chronic pathology.

Different types of CSPGs are expressed by different cell types and include versican, aggrecan, phosphocan, brevican, and others (Bradbury and Burnside, 2019; Tran et al., 2022). Decorin, a dermatan sulfate proteoglycan, and lumican, a keratan sulfate proteoglycan, are also expressed in the spinal cord after injury (Didangelos et al., 2016). At 8 weeks post-SCI, we see a reduction in several proteoglycans in *TLR4* null mice compared with wild-type mice including the CSPGs, aggrecan, versican and phosphacan, lumican, and decorin. These are located either mainly at the site of injury (versican, decorin, and lumican) and beyond (aggrecan and phosphacan). Notably our earlier work demonstrated that CSPGs elongate the period of inflammation after SCI (Francos-Quijorna et al., 2022). Therefore, the reduced expression of CSPG in *TLR4* null mice after SCI could contribute to shortening the chronic inflammatory period and improve outcomes.

CSPGs consist of a core protein to which are attached one or more GAG chains that are sulfated at the C4 and C6 positions of β1,3-*N*-acetyl-D-galactosamine (GalNAc) and the C2 position of 4-D-glucuronic acid (GlcA; Hussein et al., 2020). Digesting CSPG GAG chains with Ch ABC (Bradbury et al., 2002; Garcia-Alias et al., 2009; Bradbury and Carter, 2011; Tran et al., 2018; Bradbury and Burnside, 2019) or modification of the C4 sulfation (Pearson et al., 2018; Hussein et al., 2020) promotes axon growth in various experimental models. Reduction of such inhibitory CSPGs in *TLR4* null mice detected by the CS56 and other CSPG antibodies could underlie the increased sprouting that we see of serotonergic fibers in the ventral horn caudal to the lesion. Interestingly, previous work reported that the lack of MMP9 (in MMP9 null mice) results in less severe scar and reduced deposition of scar-related ECM molecules at 42 d after SCI (Hsu et al., 2008). MMP9 was shown to promote astrocyte migration and deposition of ECM molecules. The reduced expression of MMP9 we see in *TLR4* null mice at 8 weeks after SCI could therefore underlie the reduction in scar-related CSPG. This may contribute to the increased sprouting of descending 5-HT input from raphe nuclei in the brainstem, which is important for locomotor control (Saruhashi et al., 1996; Ribotta et al., 2000; Ghosh and Pearse, 2014).

Although we detect a reduction of aggrecan by Western blot in both genotypes early after injury (7 d), immunostaining showed that this is due to loss of expression at the lesion epicenter, as has been reported previously (Andrews et al., 2012). However, aggrecan immunostaining is seen both rostral and caudal to the lesion epicenter and appears to be higher in *TLR4* null mice than that in wild-type mice at 8 weeks. This may be due to the reduction in MMP9 in *TLR4* null mice at 8 weeks, as MMP9 has been reported to mediate the degradation of aggrecan (Fosang et al., 1992). TLR4 has also been shown to directly regulate the expression of MMP9 in astrocytes (Yang et al., 2019). Additionally, there is evidence that activation of nuclear NFκB can upregulate expression of MMP9 (Hozumi et al., 2001). Interestingly, this labeling of aggrecan is localized to PNN nets around motor neurons, which is further confirmed by the overlap of WFA staining. Aggrecan and WFA staining of PNNs have been described previously (Irvine and Kwok, 2018; Fawcett et al., 2019). The appearance of PNNs coincides with the termination of the period of plasticity and the consolidation of synapses, for example, during development and after injury (Pizzorusso et al., 2002; Tran et al., 2018; Fawcett et al., 2019; Sanchez-Ventura et al., 2021). This together with the increased sprouting of serotonergic fibers may contribute to the improved locomotor recovery in the chronic period after SCI.

This study indicates that TLR4 signaling appears to exert an unexpected late effect 8 weeks after SCI. This includes increase in NFκB signaling, chemokine/cytokine expression, activation of necroptosis pathways, and changes in expression of MMP9 and scar-related and PNN-related ECM molecules and is accompanied by late effects on axonal sprouting and locomotor recovery after SCI. Further studies are needed to explore the role of these candidate molecules in functional recovery. Deletion or inhibition of TLR4 at early or late time points after SCI, using small molecule inhibitors or conditional or cell type-specific knock-out mice, would provide further clarification of its role in SCI. This would also provide further confirmation that the novel findings we have observed are of relevance clinically and offer potential new therapeutic targets for treating people with chronic spinal cord injuries. The potential risk of infections by blocking TLR4 signaling can be avoided by local intrathecal delivery of TLR4 blockers in patients with closed vertebral fractures that are not exposed through the skin. This work highlights the potential for late-stage targeting of TLR4 as a potential therapy for chronic SCI and could have an important impact for the millions of people worldwide currently living with long-term disability due to a traumatic spinal cord injury.

## Ethics Approval

All procedures were approved by the McGill University Animal Care Committee (protocol 7884) and followed the guidelines of the Canadian Council on Animal Care. Animal procedures in the United Kingdom were approved by the Animal Welfare and Ethical Review Body (AWERB) of King's College London and conducted under Home Office Project License 70/8032 and PEE6F3C82.

## Data Availability Statement

Complete source data are provided with this paper. Data that support the findings will be made available upon reasonable request to the corresponding authors.
